# Clinical Practice Guidelines for Cannabis and Cannabinoid-Based Medicines in the Management of Chronic Pain and Co-Occurring Conditions

**DOI:** 10.1089/can.2021.0156

**Published:** 2024-04-01

**Authors:** Alan D. Bell, Caroline MacCallum, Shari Margolese, Zach Walsh, Patrick Wright, Paul J. Daeninck, Enrico Mandarino, Gary Lacasse, Jagpaul Kaur Deol, Lauren de Freitas, Michelle St. Pierre, Lynne Belle-Isle, Marilou Gagnon, Sian Bevan, Tatiana Sanchez, Stephanie Arlt, Max Monahan-Ellison, James O'Hara, Michael Boivin, Cecilia Costiniuk

**Affiliations:** ^1^ Department of Family and Community Medicine, University of Toronto, Toronto, Canada.; ^2^ Faculty of Medicine, Department of Internal Medicine, University of British Columbia, Vancouver, Canada.; ^3^ Canadian HIV Trials Network, University of British Columbia, Vancouver, Canada.; ^4^ Department of Psychology, University of British Columbia, Kelowna, Canada.; ^5^ Canadian AIDS Society, Ottawa, Canada.; ^6^ Department of Internal Medicine, Max Rady College of Medicine, University of Manitoba, Winnipeg, Canada.; ^7^ CancerCare Manitoba, Winnipeg, Canada.; ^8^ MJardin Group Canada, Toronto, Canada.; ^9^ Faculty of Pharmaceutical Sciences, University of British Columbia, Vancouver, Canada.; ^10^ Centre for Addiction and Mental Health, Institute for Mental Health Policy Research, Toronto, Canada.; ^11^ Canadian Institute for Substance Use Research, University of Victoria, Victoria, Canada.; ^12^ Arthritis Society, Toronto, Canada.; ^13^ Medical Cannabis Canada, Toronto, Canada.; ^14^ Independent Consultant, Toronto, Canada.; ^15^ CommParm Consulting, Inc., Barrie, Ontario, Canada.; ^16^ Chronic Viral Illness Service/Division of Infectious Diseases, Department of Medicine, McGill University Health Centre, Montreal, Canada.; ^17^ McGill Cannabis Research Centre, McGill University, Montreal, Canada.; ^18^ Research Institute of the McGill University Health Centre, Montreal, Canada.

**Keywords:** cannabinoids, cannabinoid-based medicines, cannabis, marijuana, chronic pain, sleep disorders

## Abstract

**Background:**

One in five individuals live with chronic pain globally, which often co-occurs with sleep problems, anxiety, depression, and substance use disorders. Although these conditions are commonly managed with cannabinoid-based medicines (CBM), health care providers report lack of information on the risks, benefits, and appropriate use of CBM for therapeutic purposes.

**Aims:**

We present these clinical practice guidelines to help clinicians and patients navigate appropriate CBM use in the management of chronic pain and co-occurring conditions.

**Materials and Methods:**

We conducted a systematic review of studies investigating the use of CBM for the treatment of chronic pain. Articles were dually reviewed in accordance with Preferred Reporting Items for Systematic Reviews and Meta-Analyses guidelines. Clinical recommendations were developed based on available evidence from the review. *Values and preferences* and *practical tips* have also been provided to support clinical application. The GRADE system was used to rate the strength of recommendations and quality of evidence.

**Results:**

From our literature search, 70 articles met inclusion criteria and were utilized in guideline development, including 19 systematic reviews and 51 original research studies. Research typically demonstrates moderate benefit of CBM in chronic pain management. There is also evidence for efficacy of CBM in the management of comorbidities, including sleep problems, anxiety, appetite suppression, and for managing symptoms in some chronic conditions associated with pain including HIV, multiple sclerosis, fibromyalgia, and arthritis.

**Conclusions:**

All patients considering CBM should be educated on risks and adverse events. Patients and clinicians should work collaboratively to identify appropriate dosing, titration, and administration routes for each individual.

**Systematic Review Registration:**

PROSPERO no. 135886.

## Introduction

Across the globe, one in five individuals live with chronic pain,^1^ and estimates of chronic pain prevalence may be 20–25% in some countries and regions.^2^ Two thirds of individuals report it as moderate to severe.^3–5^ Approximately 50% of these individuals have lived with chronic pain for more than 10 years.^3^ Chronic pain often co-occurs with insomnia, anxiety, depression, post-traumatic stress disorder (PTSD), and substance use disorders such as opioid and alcohol use disorder.^6–12^ Chronic pain and these co-occurring conditions are also among the most common conditions for which cannabinoid-based medicines (CBM), derived from the cannabis plant, are used therapeutically.^13–16^

Recently, there has been a proliferation of systematic reviews on CBM and chronic pain and co-occurring conditions. Given new legal regimes globally regarding recreational cannabis and CBM derived from the cannabis plant, health care providers need to be aware of the potential efficacy and harms. Here, we present Clinical Practice Guidelines for the use of Cannabis and CBM in the Management of Chronic Pain and Co-occurring Conditions. These guidelines examine literature focused on cannabis and CBM derived from the cannabis plant rather than synthetic, pharmaceutical-grade cannabinoids in an effort to fill an important knowledge gap.

## Materials and Methods

Our previously published protocol^17^ outlined the inclusion/exclusion criteria for studies (PICOS breakdown), process for data extraction (using Preferred Reporting Items for Systematic Reviews and Meta-Analyses [PRISMA] conventions) strategy for data synthesis, assessment of evidence and recommendations (GRADE system), risk of bias assessment, and procedures for data analysis/synthesis. Conclusions relate to availability of evidence, and the evidence available does not support doing this for every cannabinoid and cannabinoid combination.

When we report on adverse effects for specific cannabinoids (i.e., tetrahydrocannabinol [THC] vs. cannabidiol [CBD] vs. THC/CBD products), the reason is that evidence exists for these specific cannabinoids or combinations. Note that we cannot extrapolate from one cannabinoid to another, nor from a combination product to one cannabinoid, since the evidence for doing so does not exist.

Practical tips were formulated qualitatively based on a discussion of panel members from their cumulative clinical experience, rather than a formal algorithm.

### Search strategy and study eligibility

In May 2019, an electronic search was conducted for peer-reviewed articles (2001–2019), in English, in the following electronic databases: Academic Search Complete, Cochrane Database of Systematic Reviews, Evidence Based Medicine Reviews, OVID Medline, PsychINFO, PubMed, CINAHL, and Web of Science. The search strategy was as previously described.^17^ Studies focusing exclusively on efficacy of synthetic pharmaceutical cannabinoids (such as nabilone or dronabinol) were excluded.

In addition, studies reporting on results of a single patient (*n*=1) were also excluded. As nabiximols contain plant-derived cannabinoids, they were included. Complete inclusion and exclusion criteria are listed in Supplementary Table S1 in the protocol.^17^ Study types including systematic reviews, meta-analyses, controlled trials, uncontrolled trials, observational studies, and cross-sectional designs were included, whereas systematic reviews and original research articles were summarized separately. Guideline development was primarily based on original research given the heterogeneity of previous systematic reviews.

### Study screening and selection

Using the PRISMA conventions,^18^ a Data Synthesis Committee determined study eligibility by reading identified abstracts. Each abstract was independently dually reviewed. Full-text screening occurred in cases of uncertainty. Disagreements on inclusion were resolved through a discussion by two reviewers. Full-text screening was also independently dually reviewed. Reference lists of included studies were searched for potential additions. A PRISMA flowchart outlines this process (Supplementary Appendix SA1).

### Data extraction

Data were extracted independently using a standardized Data Extraction Form to create evidence tables. For each study, data were extracted related to study identification (author, year published, number and location of centres, funding, journal name), number of participants, form of CBM, dose and route, study design and setting, inclusion/exclusion criteria, aggregate demographic (age, sex, type of pain, co-occurring conditions) and clinical characteristics (co-morbidities), outcome measures, and results. We also identified types and frequencies of adverse events. Final evaluation of study quality (very low, low, moderate or high) included considerations of limitations, inconsistencies, indirectness, and imprecision.

### Data synthesis

Data were extracted using standard data extraction tools. There is significant variability in cannabis research due to heterogeneity of sample populations, study types and lengths, and CBM interventions (e.g., CBM type, dosing, administration route, etc.). Patterns related to efficacy, safety, and tolerability were explored through narrative synthesis.^19,20^ Data were compiled based on availability of quality evidence. Consistent findings and discrepancies were discussed. Evidence for CBM in the management of chronic pain and co-occurring conditions related to efficacy, tolerability, safety, indications, dosing, drug interactions, adverse events, negative effects, and contraindications.

### Assessment of evidence and recommendations

Clinical recommendations and indications were developed and presented in relation to CBM use for chronic pain and comorbidities. The Task Force used the GRADE system to rate quality of evidence and strength of recommendations.^21–26^ *Values and preferences* and *practical tips* have also been provided to support clinical application.

### Strength of recommendations

Strength of recommendations was specified as strong or weak based on risk and benefit of CBM in the specific condition. Patient values and preferences, magnitude of effect, and confidence in the evidence were considered. A recommendation was deemed to be strong if the committee considered the benefit to clearly outweigh the risk for most individuals. All other recommendations were specified as weak, indicating a need to consider individual clinical circumstances, values, and preferences.

### Risk-of-bias assessment

Two reviewers (M.S.P. and P.W.) assessed potential bias and discrepancies were adjudicated by the Data Synthesis Committee (C.C., Z.W., S.M.). The National Institutes of Health risk-of-bias assessment tools^27^ were used to assess study quality. These tools were developed specifically for different study design types, and therefore heterogeneity of the designs of included studies will not affect ability to assess quality appropriately. Study bias was graded as either “good quality” (score of 3), implying low risk of bias, “fair quality” (score of 2) implying some risk of bias, or “poor quality” (score of 1), implying high risk of bias. Assessments of bias were performed at the overall study level.

## Results

We identified 4989 records following the removal of duplicates (Supplementary Appendix SA1). Following abstract review, 4824 records were excluded, resulting in full-text review of 165 articles. Reasons for exclusion are presented in Supplementary Appendix SA1. Seventy studies were included in the final review, including 19 systematic reviews (Supplementary Appendix SA2) and 51 original research studies (Supplementary Appendix SA3). To avoid redundancy, systematic reviews were considered independent of primary literature.

### Characteristics of systematic reviews

Details of the 19 included systematic reviews are presented in Supplementary Appendix SA2, along with each review's quality rating as an assessment of risk of bias. Most reviews were rated as either *good,* or *fair*, representing low or moderate risk of bias, respectively. Included reviews were published between 2007 and 2018, with 16 of 19 reviews published in 2015 or more recently. The majority of reviews (12) included only randomized controlled trials (RCTs).

Three reviews included only systematic reviews,^28,29,30^ three contained primary research,^31–33^ and one included other systematic reviews, in addition to primary research.^34^ People with chronic pain were the most commonly studied population (14). One review focused on rheumatic disease-associated pain,^35^ one included studies of multiple sclerosis (MS) or comparable neuropathic pain,^36^ one included systematic reviews that analyzed cannabinoids for pain, spasticity, or nausea and vomiting,^29^ and two included people using medical cannabinoids regardless of indication.^32,37^

Most reviews explored both pharmaceutical synthetic and plant-based cannabinoids (15/19). Three reviews focused exclusively on plant-based cannabis and plant-derived cannabinoid extracts.^34,38,39^ Park and Wu focused exclusively on non-synthetic cannabis, as their review solely included people using medical cannabis. Reviews that focused exclusively on pharmaceutical synthetic cannabinoids were excluded.

In terms of efficacy for chronic pain reduction, most reviews (14/19) reported that cannabinoids provided analgesia in at least some contexts. Nugent et al found non-synthetic cannabinoids beneficial for neuropathic pain, but they found insufficient evidence in other types of pain. In addition, Stockings et al reported nabiximols to be effective for MS-related pain; however, generally there was insufficient evidence for cannabinoids to be used as a pain treatment. Five reviews found inadequate evidence to support cannabinoids as an effective pain treatment.^28,29–31,35^

Of the eight reviews that obtained a “good” quality rating, representing the lowest risk of bias, seven found cannabinoids to be beneficial for pain relief. However, in some instances, the analgesic effect of cannabinoids was described as “moderate”^40^ or “small.”^41^

Most reviews analyzed prevalence of adverse events associated with cannabinoids. Although common, adverse events were typically mild to moderate in severity.^34,39–42^ Adverse events were similar between reviews and included drowsiness, dizziness, and dry mouth. Aviram and Samuelly-Leichtag suggested that adverse events could not entirely be attributed to cannabinoids as “the participating patients in the included trials had pre-existing diagnoses and in many of the trials, they used concomitant medications.”^43^

A few reviews included analysis of pain comorbidities. Of the reviews that studied sleep and sleep problems within the context of chronic pain, analyses fairly consistently supported that cannabinoids provided at least partial benefit.^32,33,35,37,40–42,44,45^ Findings were more heterogenous regarding the efficacy of cannabinoids for mood disorders and related issues. Four reviews reported that cannabinoids improved anxiety in pain populations.^35,37,41,44^

Stockings et al did not find any significant difference between cannabinoid and comparator groups in overall emotional functioning or depressive or anxiety symptoms. Whiting et al reported significant improvement in anxiety with cannabinoids over placebo; however, in studies where anxiety was studied as a secondary outcome, they found no significant difference. Martin-Sanchez et al analyzed mood disturbances only within the context of adverse events but found euphoria to be a common occurrence with a number needed to harm (NNH) of 8, and dysphoria to be less common with an NNH of 29.

Finally, in a review focused on people living with chronic neuropathic pain, Mucke et al reported cannabinoids to be more efficacious than placebo for treating psychological distress.

## Evidence summaries and clinical guideline recommendations

### 1. CBM use for people with chronic pain

Forty-seven studies relevant to pain management were reviewed, including 22 RCT,^46–67^ 11 pre-post studies or uncontrolled trials,^68–78^ 11 cross-sectional or observational cohort studies,^79–89^ and 3 case series.^90–92^ Most studies (38/47) reported at least moderate benefits of CBM for chronic pain,^46–48,50,51,54,55,57,59–70,73–80,82–84,86–92^ seven were inconclusive or found insufficient evidence,^49,53,56,58,71,72,85^ and two reported mixed results.^52,81^

Associated improvements in secondary outcomes, including QoL, functionality, and mood, have also been observed with the use of medical cannabis in addition to reductions in pain severity, intensity, and interference. For details of the individual studies, see Appendix A in Supplementary Data.

#### Recommendations

1. We recommend the use of CBM as monotherapy, replacement, or adjunct treatment, in people living with chronic pain, for the management of chronic pain including central and/or peripheral neuropathic pain to improve pain outcomes.

*Strong Recommendation, Moderate-Quality Evidence*

2. We recommend the use of CBM as monotherapy, replacement or adjunct treatment, in people living with chronic pain, for mobility in those not achieving adequate response to other modalities.

*Weak Recommendation, Low-Quality Evidence*

##### Values and preferences

The recommendations place high value on the improvement in chronic pain, functionality, and secondary outcomes, including time to sleep, quality of sleep, anxiety, and depression, in those living with chronic pain and using CBM compared with placebo. The recommendations also outweigh the risks of non-serious adverse events with CBM (dizziness, disturbance in attention, somnolence, dry mouth, nausea, diarrhea) as compared with adverse events from standard analgesia (opioids and serotonin-norepinephrine reuptake inhibitors [SNRIs] or opioids monotherapy), including constipation, loss of appetite, unclear mentation, reduced affect, hemorrhoids, and substance use disorder.

##### Practical tip

A wide variety of formulations and routes including smoking, vaping, oral capsule, oral oil, and oromucosal sprays showed benefits in chronic pain, mood disorders, mobility, and sleep. Adverse events due to combustion, and exposure to second-hand smoke, make smoked CBM less favorable. The inhalation route of CBM is restricted to the vaporization of dried flower over combustion for the management of breakthrough pain due to rapid onset and shorter duration of action.

Oral products, as a route of administration may be preferred for the longer duration of action (6–8 h) compared with inhaled cannabis, particularly in the evening (due to somnolence). This will allow for greater symptom control in patients who experience chronic persistent daily symptoms and/or disease. Oral oil and capsule formulations can be easier to dose accurately (mg) and provide consistent and reproducible dosing.

##### Practical tip

The strongest evidence for reduction of chronic pain symptoms is for THC formulations, not CBD. The majority of adverse events are associated with THC. Adverse Events due to CBM are mild and may be better tolerated than other centrally acting prescription medications. Patients report that adverse effects generally subside within 48 h or when the titration phase is stopped. The best way to reduce the potential adverse effects with THC is with safe, low-dose initiation and titration (Fig. 2). In addition, a CBD dominant product can be used in combination with THC to attenuate side effects. The initiation of CBD during daytime with the addition of THC at bedtime can also further mitigate these effects.

### 2. CBM use for people with HIV and chronic pain

Three studies examined CBM to treat pain in people with HIV, including two RCTs^46,51^ and one cross-sectional study.^88^ All three studies reported significant improvements in HIV-related pain management associated with CBM use. For details of the individual studies, see Appendix B in Supplementary Data.

#### Recommendations

1. We recommend the use of CBM for the management of muscular and neuropathic pain in people living with HIV who are not achieving adequate response, or those experiencing adverse effects to other treatment modalities.

*Strong Recommendation, Moderate-Quality Evidence*

2. We recommend CBM use for the management of HIV-related symptoms, including nausea, anxiety, depression, lack of appetite, and weight loss in people living with HIV. CBM use is for symptom management only and should not replace the use of antiretroviral therapies.

*Strong Recommendation, Low-Quality Evidence*

##### Values and preferences

The recommendations place high value on the benefit of neuropathic and muscular pain relief in people with HIV over the risks of adverse events of a mainly non-serious nature such as dizziness, disturbance in attention, balance disorder, somnolence, dry mouth, nausea, diarrhea, fatigue, or confused state and those of a more serious nature such as pulmonary and cardiovascular effects of inhaled substances.

##### Practical tip

A wide variation is seen in the dose and administration routes of cannabinoids used to optimize the treatment effect and adverse event ratio. A slow dose titration, initiated with CBD-predominant cannabinoids, should be used to individualize treatment (Fig. 2).

##### Practical tip

Although benefit was observed in HIV nerve and muscle pain mostly with smoked formulations, oils and capsules have been shown to have the strongest evidence in MS. Oils and capsules provide the greatest consistency for dosage and titration and are not associated with potential adverse events associated with inhalation of CBM.

### 3. CBM use for people living with multiple sclerosis and chronic pain

Twelve studies examined CBM for pain in people with MS, including nine RCTs,^50,52,53,55,58,65,66,67,77^ two open-label studies,^77,78^ and one cross-sectional study.^82^ Seven studies involved nabiximols,^49,52,53,55,58,77,78^ three involved extracts delivered through capsule,^65–67^ and two involved whole plant cannabis.^50,82^ Study length ranged from 3 days to 2 years. Most studies (9/12) reported improvements in pain associated with CBM use, including both studies involving whole plant cannabis and all three involving extracts delivered through capsules.

The majority of studies were limited by small numbers of participants, short duration of treatment, and some crossover and blinding deficiencies. For details of the individual studies, see Appendix C in Supplementary Data.

#### Recommendations

1. We recommend the use of CBM, as adjunct treatment, for pain management in people with MS not achieving adequate response to other modalities.

*Strong Recommendation, Moderate-Quality Evidence*

2. We recommend the use of CBM, as adjunct treatment, for the management of muscle spasm in people living with MS in those not achieving adequate response to other modalities.

*Strong Recommendation, Moderate-Quality Evidence*

3. We recommend the use of CBM, as adjunct treatment, for the management of sleep disorder in people living with MS in those not achieving adequate response to other modalities.

*Strong Recommendation, Low-Quality Evidence*

##### Values and preferences

The recommendations place high value on the benefit of pain, spasticity, and sleep disturbance relief seen in people with MS over the risks of adverse events, of a mainly non-serious nature, including dizziness, disturbance in attention, balance disorder, somnolence, dry mouth, nausea, diarrhea, fatigue, or confused state.

##### Practical tip

A wide dose variation of cannabinoids was used in studies involving MS patients. Total daily THC oral dose of 10–15 mg, as a divided dose twice daily, was most commonly used. A slow dose titration should be used to individualize treatment (Fig. 2).

##### Practical tip

Although benefit was observed in pain, spasticity, and sleep with oral oil, capsule, smoked and oromucosal formulations, the strongest evidence is for the use of oils and capsules. These formulations also provide the greatest consistency for dosage and titration and are not associated with potential adverse events associated with inhalation of CBM.

### 4. CBM use for people living with an arthritic condition experiencing chronic pain

One RCT,^48^ one pre-post survey,^70^ and one published abstract^93^ have been identified in the literature search. Both the RCT and published abstract demonstrated improvement in pain in patients with an arthritic condition. For details of the individual studies, see Appendix D in Supplementary Data.

#### Recommendation

1. We recommend the use of CBM, as adjunct treatment, for the management of chronic pain in people living with arthritic conditions in those not achieving adequate response to other modalities.

*Strong Recommendation, Low-Quality Evidence*

##### Values and preferences

The recommendation places high value on the benefit of improvement in pain, sleep, and other co-morbid conditions over the risks of adverse events of a mainly non-serious nature such as dizziness, disturbance in attention, balance disorder, somnolence, dry mouth, nausea, diarrhea, fatigue, or confused state.

##### Practical tip

Best evidence of benefit is in participants with rheumatoid arthritis for improvement in pain, sleep, other co-morbid conditions, and markers of inflammation. A balanced THC/CBD oromucosal product titrated to ∼15 mg of each component may be tried. If an oral formulation is used, a slow titration of THC as shown in Figure 2 should be employed.

##### Practical tip

As dizziness and falls have been identified as potential adverse events associated with CBM use, a clear understanding of risks should be achieved before CBM initiation, especially for populations with an increased risk of bone loss/osteoporosis. Consider a lower THC starting dose, slower titration period, and consistent monitoring.

##### Practical tip

A single abstract publication has suggested benefit from topical CBD 125 mg bid for localized pain management of knee osteoarthritis. This format can be applied to the affected joints as a cream, oil, or spray. This approach can be expected to be associated with a very low risk of any adverse events. More research is needed regarding the efficacy and safety of topical CBM.

### 5. CBM use for people living with fibromyalgia and chronic pain

There was a total of six studies that included participants with fibromyalgia and pain. Four of these studies included people with fibromyalgia as part of the study participants but the authors did not produce a separate analysis for pain management in fibromyalgia and therefore these studies are not heavily considered in this section.^72,76,91,92^ Each of these studies found improvements in pain across their wider study sample. In an open-label study, two thirds of study participants living with fibromyalgia responded well to sublingual THC treatment.^72^ For details of the individual studies, see Appendix E in Supplementary Data.

#### Recommendation

1. We recommend the use of CBM, as adjunct treatment, for management of back pain, fibromyalgia pain, or other chronic pain in people with fibromyalgia who are not achieving an adequate response to standard analgesics.

*Strong Recommendation, Low-Quality Evidence*

##### Values and preferences

This recommendation places high value on the improvement in back pain, chronic pain, fibromyalgia pain, and improvement in function, quality of life (QOL), and secondary outcomes, including anxiety, among patients living with fibromyalgia from CBM use, over the low risk of non-serious adverse events (reddening of the eyes, increased appetite, and sore throat) as compared with adverse events with standard analgesics (constipation, loss of appetite, lack of mental clarity, reduced affect, and hemorrhoids).

##### Practical tip

Fibromyalgia is a heterogeneous complex pain syndrome, which frequently occurs with other pain syndromes and symptom clusters. This needs to be considered when extrapolating this study information to other patients living with fibromyalgia.

##### Practical tip

In clinical practice, SNRI are used off-label as a multimodal treatment for symptoms such as insomnia, depression, and anxiety in patients suffering from fibromyalgia; likewise, CBM can treat these symptom clusters.

##### Practical tip

All patients using CBM should be educated on proper vaporization (to avoid harms of combustion/smoking) and/or cannabis oil/capsule dosing and titration regimens. To limit adverse effects while still achieving pain outcomes, THC dosing should be started low and titrated slowly to an individual response. For elderly and frail patients, consider a lower THC starting dose, slower titration period, and consistent monitoring.

### 6. CBM use for people experiencing chronic headache and migraine

Four included studies specifically measured associations between CBM and chronic headache or migraine.^70,88,94,95^ One study was a conference abstract and included as gray literature.^94^ Of these four studies, two utilized pre/post designs,^70,94^ and two were cross-sectional.^88,95^ Each study reported at least some improvement from cannabis in participants experiencing headaches. For details of the individual studies, see Appendix F in Supplementary Data.

#### Recommendation

1. We recommend the use of CBM, as an adjunct treatment, for the management of chronic migraine or chronic headache, in those not achieving adequate response to other modalities.

*Weak Recommendation, Low-Quality evidence*

##### Values and preferences

The recommendations place high value on the benefit of migraine and headache relief over the risk of adverse events, which are mainly non-serious in nature and include somnolence and dizziness.

##### Practical tip

Some people also experience headache due to cannabis, therefore it will be important to assess the individual treatment response. Dosage form will be important to consider if CBM is used for prophylaxis versus treatment of migraine or headache, given different timing of onset.

##### Practical tip

To limit exposure to adverse events, individuals should *start low, go* slow, and follow a structured initiation and titration plan (Fig. 2). Patients and physicians should work collaboratively to identify appropriate administration route(s) that meet the needs of the individual.

### 7. CBM use for people living with chronic pain and nausea

Five studies analyzed participant perceptions of cannabis as a potential treatment for nausea as a comorbidity of pain.^79,85,88,91,92^ Each study involved whole plant cannabis in inhaled or oral formats, with patients reporting moderate relief. Although these five studies found improvements in nausea with cannabis use, other studies found nausea to be an adverse event associated with cannabis use among some participants.^46–50,54,57–59,71,72,74,77,78^

These studies were limited by study design (case series or cross-sectional surveys), small numbers of patients, unknown duration or dosing of cannabis, and the possibility of selection and recall bias. It is also unclear which cannabis formulation or route of administration is optimal (smoked vs. oral, THC vs. CBD vs. THC/CBD products) for nausea. For details of the individual studies, see Appendix G in Supplementary Data.

#### Recommendation

1. We recommend considering the use of CBM to reduce nausea in people living with chronic pain as monotherapy or adjunct treatment for those not achieving adequate response to other treatment modalities.

*Weak Recommendation, Low-Quality Evidence*

##### Values and preferences

This recommendation does not refer to the use of CBM in cancer-related pain or emetogenic therapy, and places high value on the benefit of CBM for nausea relief, over the risk of adverse events, which are mainly non-serious in nature.

##### Practical tip

All published data on nausea benefit in chronic pain have come from survey or cross-sectional studies. As such, no practical tips on cannabis type, mode of administration, or concentration of THC or CBD can be made.

### 8. CBM use for people with sleep problems and symptoms of sleep deprivation experiencing chronic pain

Sleep issues are often a target symptom for cannabis use and 25 of the studies assessed impacts of CBMs on sleep, including 16 RCTs,^47–49,52–55,57–60,65–67,74,75^ four cross-sectional survey studies,^79,80,82,87^ three pre-post style studies,^70,73,85^ and two case series studies.^91,92^ Of these studies, 10 included consumption of whole plant cannabis by participants,^60,70,73,79,80,82,85,87,91,92^ 12 involved cannabis extracts (CEs) consumed by oromucosal spray,^47–49,52–55,57–59,74,75^ and 3 involved participants treated with cannabis extract capsules.^65–67^ Almost all studies found benefit for sleep in some or most participants. For details of the individual studies, see Appendix H in Supplementary Data.

#### Recommendation

1. We recommend the use of CBM as monotherapy, replacement or adjunct treatment, to improve sleep and symptoms of sleep deprivation in people living with chronic pain not responsive to, or intolerant of, other modalities or pharmacologic treatment.

*Strong Recommendation, Moderate-Quality Evidence*

##### Values and preferences

The recommendations place high value on the benefit of CBM for disturbances in sleep and poor sleep quality. Adverse events such as somnolence, drowsiness, and sleepiness rarely caused withdrawal from studies and point to the potential benefits for patients with chronic pain states.

##### Practical tip

Many studies used CBM in the form of standardized-dose CE (nabiximols and others), which allowed for easier titration and effective dose-finding. The presence of products, including CBD, may reduce the incidence of psychiatric or euphoric effects. Oral formulations may also be considered and are not associated with potential adverse events associated with inhalation of CBM.

##### Practical tip

Sleep disturbances in patients with MS showed the most improvement with CBM used for pain and/or spasticity. This is a group who should be routinely assessed for the suitability of treatment with CBM.

##### Practical tip

To limit exposure to adverse events, individuals should *start low, go* slow, and follow a structured initiation and titration plan (Fig. 2).

### 9. CBM use for people living with chronic pain experiencing appetite loss

Seven studies assessed CBM use on appetite in participants experiencing chronic pain, including two RCTs,^59,63^ two cross-sectional studies,^79,88^ two case series,^91,92^ and one pre-post study.^70^ The RCT assessed did not demonstrate a significant difference between CBM and placebo. The two case series and one cross-sectional study reported some improvement in appetite, whereas another case series did not find significant benefit. For details of the individual studies, see Appendix I in Supplementary Data.

#### Recommendation

1. We recommend the use of THC-dominant cannabis for people with problematic loss of appetite in association with chronic pain, over no treatment.

*Strong Recommendation, Low-Quality evidence*

##### Values and preferences

The recommendation places high value on the benefit of improved appetite, and presumably nutrition, over the risks of adverse events of a mainly non-serious nature such as dizziness, disturbance in attention, balance disorder, somnolence, dry mouth, nausea, diarrhea, fatigue, or confused state.

##### Practical tip

All published data on appetite improvement in chronic pain have been associated with the use of THC dominant products. A single RCT failed to demonstrate benefit from CBD; however, this cannabinoid may provide other benefits, including a reduction in pain and anxiety.

##### Practical tip

One cross-sectional study noted an increased risk of problematic cannabinoid use in patients using CBM for appetite improvement. All patients using CBM should be monitored for cannabis use disorder. The Cannabis Use Identification Test- Revised may be used for this purpose. To limit exposure to adverse events, individuals should follow a structured initiation and titration plan (Fig. 2). Patients and physicians should work collaboratively to identify appropriate administration route(s) that meet individual needs.

### 10. CBM use for people with chronic pain experiencing PTSD

Two studies included analysis of cannabis as a treatment for PTSD symptoms.^70,79^ One study evaluating cannabis in patients with PTSD as the primary condition rated the mean helpfulness of CBM in the moderately helpful range^79^ Among the larger sample (*n*=186), higher levels of traumatic intrusions were associated with higher perceived helpfulness of cannabis.^79^ The second study found that the numbers of participants with PTSD reporting severe pain at baseline dropped from 56% at baseline to 11% at 4 months of CBM use. Improvements were also reported in ability to cope with pain and overall QOL, mood, sleep, and concentration.^70^ For details of the individual studies, see Appendix J in Supplementary Data.

#### Recommendation

1. We recommend the use of CBM to improve PTSD symptoms in people living with chronic pain not responsive to, or intolerant of, non-pharmacologic treatment.

*Weak Recommendation, Low-Quality Evidence*

##### Values and preferences

The recommendations place high value on the benefit of CBM for pain, intrusion symptoms, sleep disturbance, and improved mood and QOL seen in people living with PTSD over the risks of adverse events of a mainly non-serious nature such as dry mouth, disturbance in attention and memory, as well as the potential for the development of non-medical use.

##### Practical tip

The single study that reported dose suggested that 1–1.5 g/day of herbal cannabis was typical. However, other modes of administration were not discussed but might nonetheless be advantageous; oils and capsules provide the greatest consistency for dosage and titration, are not associated with potential adverse events associated with inhalation of CBM, and may also offer advantages in the context of sleep disturbance and nightmares that are prominent among some individuals with PTSD.

### 11. CBM use for people living with chronic pain experiencing anxiety

Eight studies within this review examined the treatment of anxiety with CBM in people living with chronic pain.^55,61,70,76,96–99^ Although these studies utilized a variety of CBM approaches, most evidence—including the relatively higher quality studies—reported anxiolytic effects of cannabis. For details of the individual studies, see Appendix K in Supplementary Data.

#### Recommendation

1. We recommend the use of CBM as adjunct therapy to improve symptoms of anxiety in people living with chronic pain not responsive to, or intolerant of, non-pharmacologic treatment.

*Strong Recommendation, Moderate-Quality Evidence*

##### Values and preferences

The recommendations place high value on the benefit of CBM for anxiety symptoms over the risks of adverse events of a mainly non-serious nature such as dry mouth, disturbance in attention and memory, as well as the potential for acute transient increases in anxiety and panic.

##### Practical tip

The only RCT to compare doses of herbal cannabis suggested that chemovars (strains) with 9% THC—which would be generally considered a moderate to low strength herbal cannabis—are more effective than herbal cannabis with low to very low levels of THC, regardless of CBD content. To limit exposure to adverse events, individuals should follow a structured initiation and titration plan (Fig. 2).

##### Practical tip

Other modes of administration might also be advantageous; specifically, orally ingested CBMs provide the greatest consistency for dosage and titration, are not associated with potential adverse events associated with inhalation of CBM, and may also offer advantages in the context of sleep disturbance that may be prominent among some individuals with problematic anxiety.

##### Practical tip

It is well recognized that THC has the potential to trigger acute transient increases in anxiety and panic. Individuals should be warned of this adverse effect and closely followed for it.

### 12. CBM use for people living with chronic pain experiencing depression

Findings for depressive symptom outcomes appeared to be contingent on the types of CBMs used, with herbal cannabis appearing to be more effective than extracts. An RCT of smoked cannabis found that it improved depressive symptoms significantly with a medium-sized effect over placebo.^60^ Three cross-sectional studies also report antidepressant effects of CBM.^82,97,98^ Studies involving the use of cannabis extracts are generally less positive regarding the benefits of CBM for treating depression in people with chronic pain. In contrast to these positive results, three RCTs found no significant difference between nabiximols and placebo in depression scores.^53,55,58^ For details of the individual studies, see Appendix L in Supplementary Data.

#### Recommendation

1. We recommend the use of CBM as adjunct therapy to improve symptoms of depression in people living with chronic pain experiencing unsatisfactory results from standard treatment.

*Weak Recommendation, Moderate-Quality Evidence*

##### Values and preferences

The recommendations place high value on the benefit of CBM for depressive symptoms over the risks of adverse events of a mainly non-serious nature such as dry mouth, disorientation, and disturbance in attention and memory. The CBM should not take the place of other anti-depressant treatments, including pharmacological and non-pharmacological treatments, such as psychotherapeutic intervention.

##### Practical tip

The only RCT to compare doses of herbal cannabis suggested that chemovars with 9% THC—which would be generally considered a moderate to low strength herbal cannabis—are more effective than herbal cannabis with low to very low levels of THC. To limit exposure to adverse events, individuals should follow a structured initiation and titration plan (Fig. 2).

##### Practical tip

Other modes of administration might also be advantageous; specifically, orally ingested CBMs provide the greatest consistency for dosage and titration, are not associated with potential adverse events associated with inhalation of CBM, and may also offer advantages in the context of sleep disturbance that may be prominent among some individuals with depression.

### 13. Adjunctive CBM use for people living with chronic pain experiencing unsatisfactory analgesia from opioid treatment

Four studies specifically addressed the efficacy of combined opioid analgesics with cannabis therapy for chronic pain. One study evaluated the addition of vaporized cannabis to patients taking sustained-release opioids for a variety of chronic pain conditions.^68^ These studies demonstrated a reduction in pain with the addition of CBM to their opioid regimen. Several additional studies found improvements in chronic pain associated with CBM use within samples that included participants concurrently using opioids to treat pain.^52,54,60,100,101^ For details of the individual studies, see Appendix M in Supplementary Data.

#### Recommendation

1. We recommend the use of CBM, as adjunctive treatment to opioids, for the management of chronic pain in those experiencing unsatisfactory analgesia from opioid treatment.

*Strong Recommendation, Moderate-Quality Evidence*

##### Values and preferences

The recommendations place high value on the improvement in chronic pain, fibromyalgia pain, functionality, spasms, and secondary outcomes of depression and anxiety, in those with chronic pain using CBM adjunctly over the low risk of non serious adverse events (fatigue, sedation, impairment in concentration, reduced salivation) as compared with adverse events with standard opioid analgesic therapy (constipation, loss of appetite, feeling of reduced mental acuity and flat affect, hemorrhoids, dependency, respiratory depression).

##### Practical tip

Evidence included in this review found efficacy of inhaled CBM as adjunct treatment for chronic pain. Inhalation as an administration route is advantageous for managing break-through pain due to rapid onset and shorter duration of action. Other routes of administration, including oils and capsules, may be preferred due to dosage consistency and lack of adverse events associated with inhalation, though further research with these routes is needed. Pain management can be individualized above baseline and can be managed on demand and titrated to desired effect.

##### Practical tip

Cannabis is rarely used as a first-line agent. It is important to assess and document the response to currently approved medications. This includes medication name, dose, duration, response, and tolerability. Some physicians will use cannabis in conditions where other treatment options have failed or are not tolerated.

##### Practical tip

Concomitant analgesics can be tapered or discontinued once a stable CBM dose is established. This may lead to a reduction in polypharmacy, side effects, drug interactions, as well as an improvement in adherence and cost saving.

##### Practical tip

As there was no change in plasma opioid levels after exposure to cannabis, improved analgesic response in patients using cannabis and opioids is likely due to synergy or additive effect. More research is required especially regarding whether cannabis should be considered as a treatment option before opioid initiation based on the lower risk of adverse effects versus opioids.

##### Practical tip

Long-term pain control was observed through a reduction in pain scores. Tolerance to adverse effects occurs within 48 h of a THC dose increase; however, tolerance to benefits did not develop over time. Starting at a low dose and gradually titrating to the lowest effective dose without adverse effects is suggested (Fig. 2).

### 14. CBM use and opioid sparing for people using opioids as treatment for chronic pain

Opioid sparing, or the reduction of opioid use, resulting from the use of CBM for pain management was the primary outcome in one study^86^ and a secondary outcome in six studies.^69,73,81,84,87,91^

Three studies found positive associations between medical cannabis use and opioid sparing.^69,73,102^ Two other studies reported that the majority of participants reduced their routine pain medications by 60–70%; however, the extent to which opioid medications were specifically reduced was unclear.^87,99^ For details of the individual studies, see Appendix N in Supplementary Data.

#### Recommendations

1. We recommend the use of CBM as adjunct treatment among people using moderate/high doses of opioids (>50 morphine equivalent) for the management of chronic pain and/or to increase opioid sparing.

*Strong Recommendation, Moderate-Quality Evidence*

2. We recommend the use of CBM as adjunct treatment for chronic pain among people using any dose of opioids who are not reaching chronic pain goals, are experiencing opioid-related adverse events, or display risk factors for opioid-related harm.

*Strong Recommendation, Low-Quality Evidence*

##### Values and preferences

The recommendations place high value on the reduction in the reliance on opioids with secondary outcomes improvements, including sleep, anxiety, and mood, in those living with chronic pain and using CBM as adjunct/concurrent to opioids over the low risk of non-serious adverse events (dry mouth, dizziness, increased appetite, sedation, concentration difficulties) as compared with adverse events with opioids/standard of care (constipation, loss of appetite, feeling of reduced mental acuity and flat affect, hemorrhoids, dependency, respiratory depression).

##### Practical tip

There is a physiologic rationale for coadministration of cannabis and opioids, which prevents opioid-tolerance and the need for dose escalation. In addition, cannabis can treat the symptoms of opioid withdrawal, reduce or replace opioids. Thus, cannabis is safer than opioids and makes opioid consumption safer. More research is required in this area.

##### Practical tip

Clinicians should re-evaluate any patient on >50 mg morphine equivalent dose (MED) as their risk of fatal overdose is doubled compared to 20 mg MED; the risk increases 10-fold with >90 mg MED. Both the United States and Canadian opioid guidelines advise clinicians to carefully reassess risk-benefit ratio when >50 mg MED and to avoid >90 mg MED as there is low evidence for improvements in pain, but a significant increase in the risk of harm.

##### Practical tip

Individuals should keep a daily log, including dosing and monitoring for efficacy, effects on mood and function, and possible side effects. This will encourage individuals to slowly titrate cannabis to symptom control, while minimizing adverse events. Once individuals using medical cannabis are stabilized, generally they do not require dose escalation over time.

##### Practical tip

Health care providers are encouraged to implement standardized self-administered questionnaires such as Patient Health Questionnaire-9, General Anxiety Disorder-7, or Brief Pain Inventory starting with the initial intake, and to continue in all subsequent follow ups to reassess risk benefit.

**FIG. 1.**
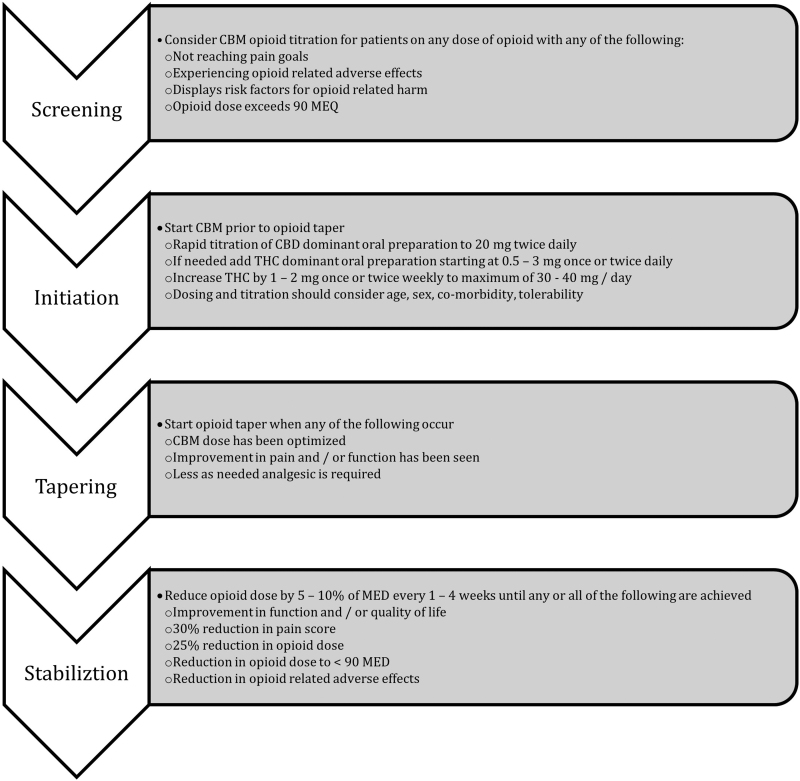
Suggested approach for adjunct cannabinoid use for opioid sparing. Adapted from Sihota et al.^103^

A Suggested Approach for Adjunct Cannabinoid Use for Opioid Sparing. Adapted from Sihota et al^103^ is shown in Figure 1.

## Additional Practical Considerations

### Drug interactions

Both THC and CBD are predominantly metabolized, by the liver, through the action of the cytochrome P450 system.^104,105^ A paucity of clinical studies are available regarding the effect of cannabinoids on this enzyme system, but *in vitro* studies suggest that THC inhibits CYP3A4, CYP3A5, CYP2C9, and CYP2C19, whereas CBD inhibits CYP2C19, CYP3A4, and CYP3A5. Due to the weak inhibitory effect of these cannabinoids, higher concentrations than those seen clinically are likely to be required for clinical inhibitory effect.^106^

This, however, may be of concern when cannabinoids are co-administered with drugs having a narrow therapeutic window and also metabolized by these enzymes, such as direct acting oral anticoagulants metabolized through CYP3A4^107,108^ and clopidogrel requiring conversion to its active metabolite by CYP2C19.^109^ Significantly elevated levels of the antiepileptic clobazam and its metabolite, n-desmethylclobazam, have been observed when co-administered with very high doses of CBD, likely due to co-metabolism through CYP2C19 and CYP3A4.^110^

Preclinical studies have reported that cannabinoids may also bind membrane transporters including breast cancer resistance protein^111^ and P-gp,^112,113^ which can, theoretically, impact the effect of other medications. A comprehensive overview of the pharmacokinetic interactions of cannabinoids has been reported by Alsherbiny and Li^114^ Pharmacodynamic interactions also need to be considered with CBM, particularly THC, administration. Additive effects can occur when cannabinoids are combined with sympathomimetics (e.g., tachycardia, hypertension), central nervous system depressants such as alcohol and opioids (e.g., drowsiness, ataxia), and anticholinergics (e.g., tachycardia, confusion).^115^

### Adverse effects

▸ A concern from patients and clinicians are the adverse effects associated with cannabis use. The adverse effects that are the most commonly associated with cannabis are related to THC-dose and the route of administration.^116^ The THC-related adverse effects include dizziness, cognitive impairment, dry mouth, tachycardia, anxiety, drowsiness, and fatigue.^116^ There is evidence to suggest a positive association between cannabis use and development of psychosis, in people susceptible to psychotic disorders.^117–119^ Although no definitive causal effects have been established, there are case reports of stroke, acute coronary syndrome, and cardiac arrhythmias associated with use of cannabis.^120,121^ Maternal exposure to cannabis can adversely affect conception and/or maintenance of pregnancy.^122–124^ Significant decline in sperm count, concentration, and motility, as well as an increase in abnormal sperm morphology have been reported.

Smoking cannabis is associated with respiratory adverse effects such as cough, an increase in phlegm, and bronchitis.^116^ Long-term use is associated with risk of cannabis use disorder, hyperemesis syndrome, as well as withdrawal symptoms including insomnia, anxiety, depression, and tremulousness.^125,126^

Adverse effects can be mitigated when initiating CBM through low-dose initiation, slow titration and avoiding smoked cannabis.^116^ In people experiencing adverse effects, clinicians can consider adjustment in the strain (chemovar) with higher CBD and lower THC, reduce the dose, or alter the route of administration to minimize these effects.^116^ The CBD can be associated with transaminitis, which is typically dose-related and more common at very high doses (800 mg/day). Transaminitis typically improves by lowering the dose of CBD. Patients using CBD regularly should undergo periodic monitoring of liver enzymes and should avoid excessive alcohol use.^127,128^

### Additional safety concerns

The CBM use is typically not recommended for children and youth.^129^ In addition, parents and those living with children should use caution to avoid exposure to second-hand smoke. People living with chronic pain and prescribing clinicians should always have a clear understanding of risks and adverse events before CBM initiation, including legal ramifications such as the inability to drive after THC consumption. It is recommended that CBM use follow a structured initiation process and have clinical supervision throughout.

### Dosing

Two publications and one poster addressing dosing of CBM have been identified.^130–132^ All are concordant with the concept of low starting dose to be titrated slowly to achieve optimal target symptom improvement with minimal off-target effects, including euphoria. The optimal therapeutic dose is the dose that allows the patient to reach treatment goals, including pain and symptom reduction and improvement in function, with minimal or no side effects. Patients do not need to feel “high” or impaired to have symptom improvement.

**FIG. 2.**
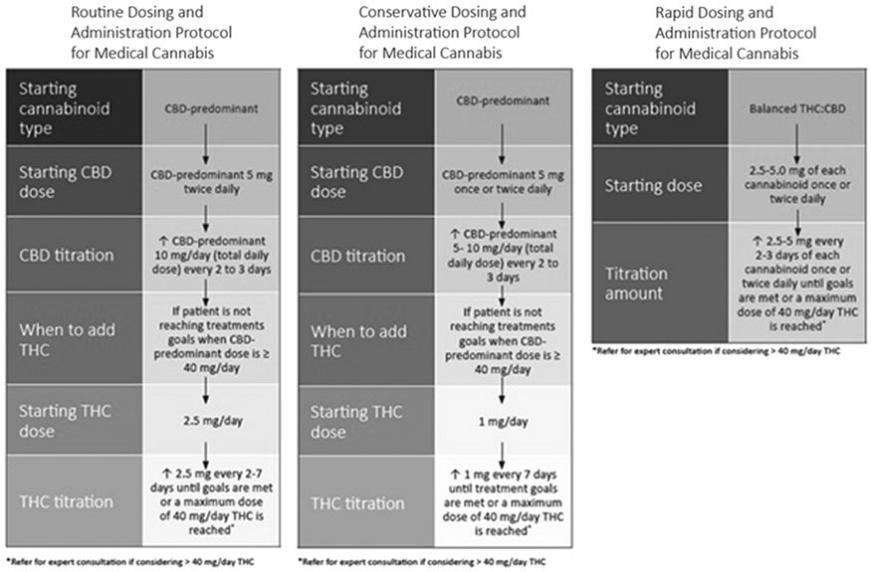
Example of oral CBM Dosing and Titration Protocols. Reproduced with permission of Bhaskar et al.^133^ CBM, cannabinoid-based medicines.

Bhaskar et al propose three dosing regimens based on the clinical situation (Fig. 2): a routine protocol appropriate for most patients, a rapid protocol for those with severe pain, terminal illness or those already taking higher dose CBM and a conservative protocol for those with frailty, severe comorbidities, and polypharmacy. PRN dosing and micro-dosing may also be acceptable for breakthrough symptom management.

### Authorization

Wide variation in legal status of medical and recreational cannabis exists globally. Clinicians authorizing or prescribing CBM should understand and comply with local laws and other regulations regarding its use. Individuals with conditions where CBM may be useful are urged to consult and be guided by regulated heath care professionals familiar with its use.

### Strengths and limitations

These guidelines fill an important gap in the literature by providing guidance for clinicians and patients during a period of cannabis regulation changes. They include a thorough systematic review with rigorous study selection and methods for data extraction, quality assessment, and data synthesis. Although CBMs may be used for other purposes, these guidelines present recommendations for people living with chronic pain and for co-occurring conditions within the context of chronic pain and may not be transferable.

From the perspectives of people with lived experience, differences between sativa-predominant and indica-predominant chemovars of cannabis are important. For example, anecdotally, THC-dominant sativa tends to be stimulating and impede sleep, can augment anxiety and increase heart rate. However, the science behind the differences between sativa-predominant and indica-predominant chemovars is not well defined and the published research is undeveloped in this area. For these reasons, they were not included in the search results that are the foundation of the article.

There exist several challenges within CBM and chronic pain research. With respect to some co-occurring conditions, there still exist relatively few controlled trials. The data related to co-morbid conditions were typically not the primary focus of included studies and, subsequently, may be underpowered. The lack of comparative studies where the safety and efficacy of CBM is compared with typical pain treatments is also problematic. In addition, challenges commonly exist with unmasking in placebo-controlled trials, representing potential risk of bias, especially as pain and many comorbidities are measured with Visual Analog Scale or other subjective measures.

## Supplementary Material

Supplemental data

Supplemental data

Supplemental data

Supplemental data

Supplemental data

## Patient and Public Involvement

Chronic pain and CBM patients were included on the guidelines task force and were involved at every stage of development, including project inception, data synthesis, guideline development, and manuscript preparation.

## Supplementary Material

Supplementary Table S1

Supplementary Appendix SA1

Supplementary Appendix SA2

Supplementary Appendix SA3

## Notes

**Cite this article as:** Bell AD, MacCallum C, Margolese S, Walsh Z, Wright P, Daeninck PJ, Mandarino E, Lacasse G, Deol JK, Freitas L, St. Pierre M, Belle-Isle L, Gagnon M, Bevan S, Sanchez T, Arlt S, Monahan-Ellison M, O'Hara J, Boivin M, Costiniuk C; and External Review Panel (2024) Clinical practice guidelines for cannabis and cannabinoid-based medicines in the management of chronic pain and co-occurring conditions, *Cannabis and Cannabinoid Research* 9:2, 669–687, DOI: 10.1089/can.2021.0156.

## Abbreviations Used

CBD: cannabidiol
CBM: cannabinoid-based medicines
CEs: cannabis extracts
MED: morphine equivalent dose
MS: multiple sclerosis
NNH: number needed to harm
PRISMA: Preferred Reporting Items for Systematic Reviews and Meta-Analyses
PTSD: post-traumatic stress disorder
QOL: quality of life
RCTs: randomized controlled trials
THC: tetrahydrocannabinol
