# Supplementary material for: Clinical Practice Guidelines for Cannabis and Cannabinoid-Based Medicines in the Management of Chronic Pain and Co-Occurring Conditions
Source: Cannabis Cannabinoid Res. 2024 Apr 1;9(2):669–87. doi: 10.1089/can.2021.0156 (PMC10998028; doi:10.1089/can.2021.0156)
Supplement: Supplemental data [file Suppl_Data.docx]

###

**Clinical Practice Guidelines for Cannabis and Cannabinoid-Based Medicines in the Management of Chronic Pain and Co-occurring Conditions**

### **Appendix A - CBM Use for People with Chronic Pain**

### Whole Plant Cannabis:

Of the 26 included studies involving whole plant cannabis consumption, 24 found beneficial results for managing chronic pain. Ten of the 26 studies included broad chronic pain populations,^1–9^ four studies involved neuropathic pain,^10–13^ three focused on people living with HIV,^14–16^ two on people living with MS,^17,18^ two on people prescribed opioids,^19,20^ two on people using medical cannabis,^21,22^ one on people living with fibromyalgia,^23^ one on people living with post-traumatic stress disorder (PTSD),^24^ and one on men living with chronic prostatitis/chronic pelvic pain syndrome.^25^ By study type, the 26 included studies involving whole plant cannabis included seven RCTs,^10–15,17^five pre/post studies or uncontrolled trials,^1–3,24,26^ 11 cross sectional or observational cohort studies,^4–6,16,18–23,25^ and three case series.^7–9^ Significant improvements in pain outcomes were produced with cannabis in each of the seven RCTs as compared with placebo. In people with chronic pain, treatment response was seen within as few as five days, however, the study duration was short.

Three studies measured the immediate effects of vaporized or smoked cannabis and each found significant reductions in pain as compared with placebo.^11–13^ One was a double-blind, placebo-controlled, crossover study of vaporized cannabis (1.29% THC; 3.53% THC) among treatment-resistant neuropathic pain patients (N = 39; 28% female), including participants with peripheral (Type I Complex Regional Pain Syndrome (CRPS), 15%; causalgia, 5%; diabetic neuropathy, 15%; idiopathic peripheral neuropathy, 8%; postherpetic neuralgia, 8%; brachial plexopathy, 8%; lumbosacral radiculopathy, 8%) and central (spinal cord injury, 23%; central neuraxis by multiple sclerosis, 8%; thalamic pain, 3%) neuropathic pain. Participants inhaled either placebo, 1.29% THC or 3.53% cannabis over two administration periods (separated by 1.5 hours) at three different sessions that were each separated by an average of seven days.^11^ Greater analgesia was reported after the second administration of either dose of active cannabis, 1.5 hours post-initial dose (p < 0.0001) and remained stable four and five hours post-initial dose (p = 0.004 and p = 0.0018, respectively).^11^ To achieve a 30% reduction in pain, the number needed to treat (NNT) were: placebo vs. 1.29% THC (NNT=3.2; p = 0.0069), placebo vs. 3.53% THC (NNT=2.9; p = 0.0023) and 3.53% THC vs. 1.29% THC (NNT=25), signifying equal analgesic efficacy amongst both cannabis doses.^11^ Both active doses produced significant decreases in deep pain, pain intensity, unpleasantness (all p < 0.0001) and sharpness (p < 0.001), with the low dose producing the greatest reductions in burning and aching pain.^11^ When adjusting the results based on the type of pain (central vs. peripheral pain), no significant effects were produced by the pain-type covariate (p > 0.8) while previous outcomes remained similar. Cannabis was well tolerated, producing minimal psychoactive effects and neuropsychological alterations that were typically dose-dependent, short in duration and subsided within one to two hours. Treatment produced significant findings on ratings of “feeling high” (p < 0.003) and “feeling sedated” (p < 0.0001) but there were no serious adverse events or participant dropout due to experimental interventions provided.^11^ Significant negative impacts on daily functioning such as psychomotor, learning and memory outcomes were not seen with 1.29% THC cannabis.^11^

In a previous double-blinded, placebo-controlled, crossover study administering smoked cannabis cigarettes (3.5% THC, 19 mg THC; 7% THC, 34 mg THC) for central and peripheral neuropathic pain (N = 38; 47% female; 32 completers), the same authors found similar results.^12^ Using a comparable dosing regimen of two administrations of either placebo, 3.5% THC or 7% THC cannabis at each of the three sessions (separated by an average of eight days), *Wilsey et al* (2008) found that active cannabis produced significant analgesic effects compared to placebo with no differences between the cannabis doses (p = 0.95).^12^ Participants with CRPS Type I (58%), central neuropathic (spinal cord injury or multiple sclerosis, 26%) and peripheral neuropathic pain (diabetic neuropathy or focal nerve injury, 16%) experienced significant reductions in VAS (visual analog scale) pain intensity per minute from both cannabis doses combined compared to placebo (mean difference: –0.0035, 95% CI: [– 0.0063,– 0.0007], p = 0.016) which did not differ by pain type (p = 0.39).^12^ Reductions in pain intensity per minute were significant for both doses compared to placebo (mean difference: -0.0050, 95% CI: [–0.0073, –0.0026], p < 0.01) suggesting that cumulative cannabis dosing produced an analgesic response that reversed one to two hours after the last dose.^12^ Pain was also found to be more tolerable at greater cumulative doses of cannabis (for both cannabis doses) compared to placebo (mean difference change per minute = –0.21, 95% CI: [–0.33, –0.09], P < .01) in addition to providing greater pain relief on a 7-point impression of change measure (mean difference: 0.12, 95% CI: [0.064, 0.18], P < .01).^12^ Both active doses also significantly decreased sharp (p < 0.001), burning (p < 0.001), aching (p < 0.001), sensitive (p = 0.03), superficial (p < 0.01) and deep pain (p < 0.01)compared to placebo on a neuropathic pain scale, with the high-dose producing greater analgesia compared to low-dose cannabis for superficial pain only (p = 0.04).^12^ Similar to their study conducted in 2013,^11^ Wilsey *et al* noted cannabis to be well tolerated, producing minimal psychoactive effects and some dose-dependent acute cognitive effects, most notably memory.^12^ Greater cognitive impairment was experienced resulting from high-dose cannabis for attention, learning, memory and psychomotor speed whereas only learning and memory were impacted by low-dose cannabis compared to placebo.^12^ The most common side effects for both active doses compared to placebo included ratings of “feeling high” (p < 0.05), “impaired” (p = 0.003), “sedation” (p < 0.01), “hunger” (p < 0.001) and “confusion” (p = 0.03) of which the former two effects declined overtime.^12^ No serious adverse effects or participant dropouts due to study intervention were reported, although one participant was removed due to high blood pressure noted prior to the third session.

In a 3-day RCT, Corey-Bloom *et al* (2012) found smoked cannabis (4% THC) significantly reduced pain scores over placebo.^17^ Similarly, a study with people living with HIV found participants in the cannabis group (3.56% THC) were significantly more likely to have a 30% reduction in pain compared with placebo. Another RCT with people living with HIV found similar analgesic results when cannabis was titrated to optimum dose between 1% and 8% THC.^15^ With a crossover design Ware *et al* found significantly reduced daily pain scores when chronic treatment-resistant, post-traumatic or people living with post-surgical neuropathic pain (N = 23; 52% female; 21 completers) were treated with 9.46% THC but not at 2.5% or 6% (4 sessions [25 mg THC smoked via a pipe, 3x/day per session] lasting five days each, separated by nine days).^10^Specifically, average daily pain intensity was significantly reduced when taking 9.4% THC compared to placebo (mean difference = 0.7; 95% CI, 0.02–1.4; p = 0.023) in addition to improvements in the ability to fall asleep (p < 0.001), quality of sleep, (p = 0.01) anxiety (p < 0.05) and depression (p < 0.05).^10^ Cannabis was well tolerated, with no significant differences noted for measures of mood and quality of life. Some mild adverse effects were experienced, most commonly with 9.4%THC including headache, dry eyes, dizziness, cough and feeling “high” as well as some moderate adverse events (fall, increased pain, numbness, drowsiness, pneumonia). No serious adverse effects were reported.

The five pre/post studies involving whole plant cannabis as a treatment for chronic pain consistently reported significant reductions in pain at study end compared with baseline. Three studies with chronic pain samples found significantly less pain at three months,^1^ six months,^2^ and one year.^3^ Similarly, in a sample of people living with PTSD, Chan *et al* found a significant reduction in pain at 10 months.^24^ In a short-term study with people living with chronic pain prescribed opioids, participants experienced less pain following the addition of vaporized cannabis, without significant change to plasma opioid levels.^26^

Of the 11 included cross-sectional or observational cohort studies involving whole plant cannabis for the treatment of chronic pain, three studies found moderate efficacy in participants who were also prescribed opioids.^19,20^ An additional study with people prescribed opioids for chronic pain had mixed results, with those using cannabis rating it as moderately effective (mean of 6.5/10).^4^ Two cross-sectional studies with participants using medical cannabis found analgesic effects. Eighty-seven percent reported pain alleviation in a study by Brunt *et al*,^22^ and Bonn-Miller *et al* found participants reported cannabis as moderately to mostly helpful for reducing pain.^21^ In an outpatient survey of men with chronic prostatitis/chronic pelvic pain syndrome, the majority of respondents (70%) reported that pain was slightly or much better due to cannabis use, however overall efficacy was unclear.^25^ Clark *et al* found 10/12 participants living with MS using cannabis for pain management identified it as moderately to completely helpful.^18^ In an observational crossover study with people living with fibromyalgia,^23^there was a significant reduction in pain intensity observed. Similarly, in a cross-sectional study with people living with HIV, 94% reported improvements in muscle pain and 90% reported improvements in nerve pain.^16^ A prospective, cross-sectional study of chronic pain participants found 78% reported at least moderate relief of pain (13 moderate, 11 strong and one complete relief).^23^

Data from three case series show similar moderate analgesic effects of whole plant cannabis use. In a study by Ware *et al*, 80% (N=15) of people living with chronic pain reported that cannabis improved pain.^9^ In a similar case series with people living with chronic pain, 93% reported at least moderate pain relief (greater than or equal to 6/10).^8^ Modest improvements were seen in a study by Fanelli *et al* where 76.2% of patients continued cannabis use at follow-up, of whom 64.7% reported an improvement in pain.^7^

### Oromucosal Spray:

Of the 15 studies involving chronic pain treatment with an oromucosal spray of whole plant cannabis extract, 10 suggested beneficial analgesic effects, and five found no or insufficient evidence. MS was the population type most commonly studied for this administration route, for which there were mixed findings. An RCT^27^ and an associated open-label, extension trial^28^ found a significant reduction in pain in favour of CBM that was maintained over two years. During the extension trial, participants achieved pain reduction with a mean dose of 7.5 sprays per day (2.7mg THC/2.5mg CBD per actuation). In a four-week pre/post study, nabiximols were similarly found to significantly reduce pain scores.^29^ However, four RCTs found nonsignificant differences between nabiximols treatment and placebo.^30–33^Interestingly, Langford *et al* had mixed results with a significant reduction in pain in favour of treatment at 10 weeks but not at study end (14 weeks).^31^ Collin *et al* found a nonsignificant reduction in pain, however, post hoc analysis revealed that 76% of participants who responded well in terms of spasticity (>30% improvement) also showed a >30% improvement for a variety of other symptoms including pain.^30^

An additional four studies included participants with various neurological pain conditions.^34–37^ One double-blind, parallel RCT found a significant reduction in all measures of pain and pain-related comorbidity when unilateral peripheral neuropathic pain patients with allodynia (N = 125; Intention to Treat (ITT): 63 nabiximols, 62 placebo; per-protocol (PP): 47 nabiximols, 56 placebo) remaining on their existing stable analgesic medications were treated with nabiximols via self-titration for five weeks, as compared with placebo.^34^Patients with postherpetic neuralgia (14%), peripheral neuropathy (20%), focal nerve lesion (43%), radiculopathy (10%), CRPS type II (12%) and other pain (1%) self-administered a mean (SD) number of sprays of 7.3(3.5) of nabiximols and 10.9(3.9) of placebo during the first week which remained relatively stable for nabiximols over the whole study period [10.9(6.8) sprays] in contrast to placebo [19.0(8.3) sprays].^34^ Greater reductions in pain intensity was experienced after the second week of self-titration and maintained throughout the rest of the study among those taking nabiximols compared to placebo (mean adjusted scores -1.48 points [22% reduction] vs. -0.52 points [8% reduction] on a 0–10 Numerical Rating Scale [mean difference: -0.96; 95% CI: -1.59, -0.32; p = 0.004]) in addition to improvements in sleep (p = 0.001), Pain Disability Index scores (p = 0.003), Neuropathic Pain Scale composite score (p = 0.007) and Patient’s Global Impression of Change (p < 0.001).^34^ Similar findings were reported from an analysis of the PP population (mean difference, -1.42; 95% CI: -2.10, -0.74; p < 0.001).^34^ The NNT to achieve a 30% reduction in pain scores was calculated to be 8.6 for nabiximols, with 26% of participants attaining a 30% improvement in pain intensity compared to placebo (15%).^34^ The calculated odds ratio for attaining greater efficacy when taking nabiximols compared to placebo was 3.55 (95% CI: -7.61, -1.72) in favour of nabiximols.^34^ Compared to placebo, nabiximols significantly improved dynamic (mean difference: -0.82; 95% CI: -1.6, -0.03; p = 0.042; NNT 9.2) and punctuate (mean difference: -0.87; 95% CI: -1.62, -0.13; p = 0.021; NNT 5.9) allodynia.^34^ Adverse events were mild and common in the nabiximols group (91% of participants) compared to the placebo group (77%) early on in the study with the most frequent relating to central nervous system or gastrointestinal effects (nausea, vomiting, diarrhoea, constipation). Gastrointestinal effects were significantly more common in the nabiximols group compared to the placebo group (49% vs. 32%; p = 0.003) unlike nervous system-related events (52% vs. 37%; p > 0.10).^34^ Severe nervous system adverse effects were reported among 11% of participants using nabiximols and 8% of those taking placebo. Dropouts during the study were more common among the nabiximols (18%) versus the placebo (3%)group in addition to failing to complete the study (21% vs. 11% respectively).^34^ A case of severe psychiatric adverse effects was reported both in the nabiximols and placebo groups in addition to more mild cases of psychiatric effects.^34^ A transient ischaemic attack was recorded as a serious adverse event in the nabiximols group but was specified as unrelated to experimental intervention. No differences emerged for cognitive functioning or psychomotor performance between nabiximols and placebo. Results from a 52-week open label extension study following this RCT (N = 76 participants) showed maintenance of pain relief without increased doses or toxicity.^34^

In a consecutive series of double-blind, single-patient crossover RCTs Wade *et al* found self-titration of 2.5 mg THC and 2.5 mg CBD extracts each delivered sublingually over a two-week period both produced significant reductions in pain among people living with neuropathic pain (N = 20, 50% female; multiple sclerosis, 60%; spinal cord injury, 20%; brachial plexus damage, 5%; limb amputation due to neurofibromatosis, 5%) compared with placebo on a 100 point VAS [54.6(27.4), p< 0.05, and 54.8(22.6), p < 0.05, respectively].^36^ Interestingly, when cannabinoids were administered as combined treatment (2.5mg THC/2.5mg CBD) reductions in pain did not reach statistical significance on a 100 point VAS [51.3(27.0), p > 0.05].^36^ Sublingual THC-only additionally improved muscle spasms, spasticity and appetite (all p < 0.05) whereas THC+CBD significantly improved sleep and muscle spasms (both p < 0.05).^36^ The initial open-label phase of this study included 24 participants, 4.2% of whom withdrew resulting from a vasovagal episode, another 4.2% due to sublingual burning sensations, 4.2% due to intoxication with no added benefit and 4.2% due to a failure of treatment period completion resulting from THC-induced psychoactive effects.^36^ The rapid and initial dosing of THC during the open-label phase caused transient hypotension and intoxication among some, resulting in the authors adjusting dosing to subsequent participants (from 8 sprays every two hours to 2 sprays every two hours).^36^ Adverse effects were most prominent during the open label phase and for THC-only (55%) although the proportion of participants experiencing adverse effects related to THC were similar to the placebo group (48%).^36^ The most common adverse effects included ‘drug toxicity’, headache, nausea, vomiting and sore mouth.

In an open-label add-on study from two parent RCTs, Hoggart *et al* demonstrated long-term continued efficacy (38 weeks) of self-titrated oromucosal THC:CBD (2.7 mg THC/2.5 mg CBD) spray among people living with peripheral neuropathic pain associated with diabetes or allodynia (N = 380; 234 completers; 47% female).^37^ Mean daily doses were 8.9 sprays per day and 9.5 sprays per day for allodynia RCT participants and the diabetic neuropathy RCT participants, respectively.^37^ Participants experienced a mean neuropathic pain severity reduction from 6.9 points (month one) to 5.5 points (month four) on a 11-point numerical pain scale, which at nine months continued to decrease to 4.2 points.^37^ Similar findings demonstrating continued improvement over nine months were observed among the proportion of patients reporting at least a 30% clinically relevant improvement in pain, with at least 50% of all participants reporting at least a 30% reduction in pain at all time points.^37^ Of the 28% of new responders attaining a 30% improvement in pain severity, 12% consisted of an allodynia population and 16% were a diabetic neuropathy population, of which more than half previously received placebo in their parent RCT.^37^ Associated improvements in sleep quality, global impression of change, neuropathic pain scale scores and quality of life were also experienced throughout the nine-month duration.^37^ The oromucosal cannabis spray was well tolerated with no associated adverse effects resulting from the long-term use and no dose escalations over time. For instance, the median number of daily sprays self-administered reduced from 8 sprays/day in the first month post-study initiation to 6.6 sprays/day in the ninth month post-study initiation, suggesting tolerance did not develop over time.^37^ Adverse events were typically low and mild with the most common being dizziness (19%), nausea (9%), dry mouth (8%), dysgeusia (7%), somnolence (7%) and impairment (6%).^37^ Treatment-related serious adverse events were experienced by 1% of participants and were related to nervous system and psychiatric disorders (amnesia, paranoia, suicide attempt).^37^ Study treatment was permanently ceased by 23% of participants due to adverse events, 7% resulting from severe adverse events and 18% due to treatment-related adverse events.^37^ It is worth noting that 84% of participants used concomitant analgesic medication during the study.^37^

In a double-blind, parallel RCT with people living with peripheral neuropathic pain experiencing allodynia (N = 246; 128 to THC:CBD, 118 to placebo; 173 completers; 61% female), there was a significant difference in favour of self-titrated THC:CBD spray (2.7 mg THC/2.5 mg CBD) at the 30% pain reduction clinical threshold compared to placebo (ITT dataset, OR = 1.97; 95% CI: 1.05–3.70; p = 0.034).^35^ Similar findings were observed in the PP analysis of at least a 30% improvement in pain scores with an odds ratio of 2.27 (95% CI: 1.12–4.57; p = 0.021) in favour of THC:CBD treatment (36% vs. 20% responder rates for THC:CBD vs. placebo). However reduction in mean pain scores on a 11-point numerical rating scale did not reach statistical significance over a 15-week period for either the intention-to-treat (ITT) (treatment difference: −0.34 points; 95% CI: −0.79 to 0.11 points; p = 0.14) or PP (treatment difference: −0.48 points; 95% CI: −1.08 to 0.12 points; p = 0.12) analyses despite being in favour of CBM treatment.^35^ Associated improvements were observed for all secondary endpoints directly measuring pain intensity (except for punctate allodynia) in addition to sleep quality (p = 0.0072) and Subject Global Impression of Change ratings (p = 0.023) for THC:CBD oromucosal spray compared to placebo.^35^ Participants in the CBM group were self-administering a mean daily dose of 8.9 sprays compared to 14.2 sprays in the placebo group.^35^Adverse events were mainly mild-to-moderate with the most common treatment-related effects including dizziness, nausea, fatigue and dysgeusia. ‘Psychiatric disorders’ (dissociation and disorientation) were experienced by 28% of participants in the THC:CBD group compared to 11% in the placebo group.^35^ Serious adverse events were reported by 8% of those in the THC:CBD group and were not considered treatment-related compared to the placebo group (5%), <1% of which was treatment-related.^35^ The proportion of participants ceasing medications due to adverse effects was greater in the THC:CBD group (19.5%) compared to the placebo group (6.8%).^35^ Similarly to other included studies,^34,37^ 90% of participants included in Serpell *et a*l’s^35^ study continued their current analgesic regimen throughout the study. However, in contrast to Hoggart *et al*^37^ and Wilsey *et al*,^11,12^ patients with pain related to diabetes and/or CRPS type I pain were excluded and instead only those with post-herpetic neuralgia, peripheral neuropathy, focal nerve lesion, radiculopathy or CRPS type 2 were included,^35^ similar to Nurmikko *et al*.^34^

One RCT tested the effects of two oromucosal CBM sprays (1:1 THC:CBD and THC only) with people living with brachial plexus root avulsion and found that both treatments produced significant reductions in mean pain scores compared with placebo.^38^ An additional double-blind, parallel RCT found nabiximols was associated with a significant reduction in morning pain on an 11-point pain scale during movement (treatment difference: -0.95; 95% CI: -1.83, -0.02; p = 0.044) and at rest (treatment difference: -1.04; 95% CI -1.90, -0.18; p = 0.018) for people with rheumatoid arthritis (N = 58; 31 to cannabis, 27 to placebo; 79% female) over 5 weeks of cannabis treatment delivered in the evening [mean(SD) daily dose for the cannabis group: 5.4(0.84) sprays; mean(SD) daily dose for the placebo group: 5.3(1.18) sprays].^39^ The authors suggest the decrease in pain upon movement may be representative of peripheral analgesia, compared to central effects that are associated with decreased pain at rest.^39^ THC:CBD spray administered to participants in the evening additionally produced significant improvements in pain at present the following morning (treatment difference: -0.72; 95% CI: -1.30, -0.14; p = 0.016), quality of sleep (p = 0.027) and disease activity scores (p = 0.002).^39^ However, no significant improvements were noted for morning stiffness (p > 0.4), total intensity of pain (p > 0.3) or intensity of pain at present (p > 0.5).^39^ Adverse effects were typically mild or moderate with the most common side effect being dizziness (26%).^39^ Severe adverse effects were not experienced by participants in the cannabis group compared to 7% of those receiving placebo.^39^ Withdrawals due to adverse events were reported among the placebo group (11%) but not among the cannabis group (0%).^39^

### Extract Capsules:

Three RCTs involved the treatment of pain with cannabis extract capsules for people living with MS, each finding a significant benefit in favour of CBM.^40–42^ Sample size ranged from 279 to 630, and study length from 12 weeks to one year. Results of Zajicek *et al* were somewhat mixed as a significant reduction in pain in favour of CBM over placebo was found at four and eight weeks but the reduction in pain was statistically nonsignificant at 12 weeks.^41^

### THC Oil:

Two studies involved THC treatment and had conflicting findings. In a randomized controlled, cross-over trial, Weizman *et al* found a single THC dose produced a significant reduction in ongoing pain within 15 participants with chronic radicular neuropathic pain.^43^ However, in an open-label study, five out of 13 participants reported adequate response in treating chronic pain, whereas eight reported inadequate or no response to treatment.^44^

### CBD:

The only included study exclusively focused on CBD consumption found six out of seven participants reporting improved pain scores following three weeks of treatment.^45^ However due to the short time frame, small sample size and lack of control, authors concluded more evidence was needed to determine efficacy of CBD as a treatment for pain.

### Summary:

The majority of limitations in the included studies involved issues with;blinding,^61,62,65,73,76,80,97^ the lack of control or comparison groups,^2,8,9,21–23,26,37^ study designs,^3,4,16,24,28,37^ short time periods of study,^10–13,43^ selection biases,^6,9,20^ small sample sizes,^1,9,10,44^ concomitant use of rescue medications,^35–37^ statistical biases,^35,36^ and self-reported measures.^18^ Additionally, studies were heterogeneous regarding eligibility criteria for participants including pain type, current use or history of cannabis use, hypersensitivity to cannabis and mental health disorders such as comorbid depression in addition to cardiovascular-related diseases. Therefore, further evidence is needed when considering cannabis for patients with these comorbidities.

Unfortunately there was no mention of cannabis education or individualized dosing plans, the lack of which can lead to inadequate pain relief and/or increased adverse effects. Inclusion of a structured cannabis education program (to improve patient knowledge of CBM use and risk management strategies) and individualised dosing regimens could minimize this gap in medical cannabis intervention.

Of note, a 2014 update to the Canadian Pain Society’s (CPS) evidence-based consensus statement has placed cannabinoids as a third-line analgesic agent with a combined NNT of 3.4 among moderate to severe neuropathic pain states.^46^ Gabapentinoids, tricyclic antidepressants and serotonin noradrenaline reuptake inhibitors were classified as first-line treatments whereas tramadol and controlled-release opioid analgesics were categorized as second-line treatments. Fourth-line agents include methadone, anticonvulsants with lesser efficacy (lamotrigine, lacosamide), tapentadol and botulinum toxin. The CPS concluded there is evidence to support some analgesic combination among selection of neuropathic pain conditions and that treatments should be individualized based on the patient factoring efficacy, adverse effects, accessibility and affordability.

Additionally, numerous Phase I-III trials including over 2000 patients in total (with greater than 1000 patient years of exposure) provide evidence of significant improvements from nabiximols for subjective sleep parameters (40-50% attaining good or very good sleep quality) in various pain conditions such as MS, peripheral neuropathic pain, intractable cancer pain, and rheumatoid arthritis while producing minimal adverse effects.^47^ Evidence suggests there is no need to increase doses over four years as no tolerance is observed to the benefits of nabiximols on sleep.

Based on these studies it is evident that medical cannabis may be beneficial in the treatment of chronic pain. Of the 47 included studies, 38 found medical cannabis to significantly reduce chronic pain among individuals with chronic pain using a variety of cannabis products and routes of administration. Associated improvements in secondary outcomes including quality of life, functionality and mood have also been observed with the use of medical cannabis in addition to reductions in pain severity, intensity and interference. Medical cannabis appears to be well-tolerated, with adverse effects related to its use typically mild in nature (i.e., sedation, dizziness, headache and feeling “high”). Adverse events are related to the dose of THC with a duration dependent on the route of administration. Further research should address issues with blinding and ensure adequate control or comparison groups are included as part of the study design. Additionally, further research is needed to compare safety and efficacy of CBMs and first and second line therapies, including opioids This will aid in not only reducing biases but in clarifying the extent of the effect medical cannabis has on pain when compared to placebo or a control group.

***Appendix B - CBM Use for People with HIV and Chronic Pain***

In a five-day RCT, Abrams *et al* found that smoked cannabis containing 3.56% THC, consumed three times per day for five days resulted in at least a 30% reduction in pain for 13 out of 25 participants randomly assigned to the treatment group, compared to only 6 out of 25 receiving placebo treatment.^14^ Participants receiving active treatment reported a median reduction in chronic neuropathic pain of 34% compared with 17% in the placebo group (p=0.03).

In a similar, five day, cross-over clinical trial, Ellis *et al* asked participants to self-titrate smoked cannabis to achieve the most effective reduction in pain without experiencing unacceptable adverse events (1-8% THC by weight).^15^ They found pain reduction to be significantly greater for cannabis compared with placebo. Participants were also significantly more likely to achieve a 30% reduction in pain (0.46 (95% CI: 0.28, 0.65) vs 0.18 (95% CI: 0.03, 0.32), p=0.043)) while taking cannabis than placebo (NNT=3.5).

In a cross-sectional study, Woolridge *et al* assessed common symptoms for which CBM were used, and perceived effectiveness in a sample of people living with HIV.^16^ Within the sample, 45% reported experiencing muscular pain, of which 94% reported improvement due to the use of CBM. Statistically significant improvements were also found for symptoms of neuropathic pain, lack of appetite, nausea, anxiety, depression and weight loss (all p<0.01).

The studies by Abrams *et al* and Ellis *et al* were both RCTs and included a placebo group to compare with the active treatment groups. In both studies, participants were on the treatment for only 5 days, which is a relatively short period of time. Although all individuals had prior experience with cannabis smoking, a limitation of studying smoked cannabis is that it is not possible to precisely control the amount of THC inhaled. Furthermore, both of the RCTs only examined THC, therefore it is not possible to draw conclusions about the efficacy of CBD or THC:CBD combination treatments. In the Ellis *et al* study, participants self-titrated the THC to find the best result without unacceptable side effects.^15^

***Appendix C - CBM Use for People Living with Multiple Sclerosis and Chronic Pain***

The Multiple Sclerosis and Extract of Cannabis (MUSEC) study randomized 279 participants living with MS to oral cannabis extract, containing 2.5 – 12.5 mg THC twice daily titrated to optimize benefit, or placebo.^41^ Approximately 80% of participants were taking other analgesic or antispasmodic medications. The primary endpoint of relief from muscle stiffness, as measured on an 11-point category rating scale after 12 weeks was almost twice as high with cannabis extract (CE) than with placebo (29.4% vs 15.7%; OR 2.26; 95% CI 1.24 to 4.13; p=0.004, one sided). All pre-specified endpoints, relief of body pain, muscle pain and sleep disturbance, at 4, 8 and 12 weeks also improved significantly (p<0.025) except relief of body pain at 12 weeks (p=0.028).^41^ When the pain analysis was restricted to those with higher degrees of body pain response rates were higher at all timepoints (p=0.001). The proportion of patients with adverse effects (AEs) was higher in the CE group than in the placebo group (93.0% vs 74.6%). Thirty participants in the CE group (21.0%) and nine in the placebo group (6.7%) were withdrawn from the study or discontinued study medication due to AEs. More than 95% of AEs in each treatment group were mild or moderate, and the majority subsided after the end of study/treatment. AEs that occurred at clearly higher rates in the CE group than in the placebo group (difference >3%) were dizziness, disturbance in attention, balance disorder, somnolence, dry mouth, nausea, diarrhoea, fatigue, asthenia, feeling abnormal, urinary tract infection, disorientation, confused state and fall. No relevant changes were seen in haematology, blood chemistry, urinalysis or vital signs. In contrast, the cannabinoids for treatment of spasticity and other symptoms related to MS (CAMS) study^40^ randomized 630 subjects to oral CE, THC, to a maximum 12.5 mg THC twice daily, or placebo failed to demonstrate benefit for the primary endpoint of change in overall spasticity scores, using the Ashworth scale (estimated difference 0·32 (95% CI –1·04 to 1·67). However, when the 11-point category rating scale was used, significant improvement was observed for spasticity, pain and sleep (p=0.01, 0.002, 0.025 respectively). A 12-month extension of the CAMS study^42^ demonstrated highly significant reductions in pain, spasms, spasticity, and sleep (p=0.002, 0.002, 0.004 and 0.016, respectively) at 52 weeks in the 80% of patients who remained on treatment. The authors speculated that the lack of benefit in the main study is the Ashworth scale being too insensitive to identify small but clinically significant effects on spasticity. Adverse events were similar to those observed in the MUSEC study.

Three studies found non-significant reductions in pain,^28,30,33^ all RCTs exploring the efficacy of nabiximols in participants with MS. Wade *et al* found a large reduction in pain in both placebo and treatment groups but no statistical difference between the groups.^33^ Collin *et al* did not find a statistically significant improvement in oromucosal spray CBM over placebo, however, post-hoc analysis showed 76% of spasticity responders also reported greater than 30% improvement in pain scores, supporting the potential use of CBM to treat multiple symptoms concurrently.^30^ In a large trial of participants living with MS, Novotna *et al* also found a non-significant reduction in pain.^32^ The remaining four studies involving oromucosal spray intervention all produced significant reductions in pain. Both open-label studies reported a significant reduction in pain at study end compared with baseline.^28,29^ In a 14-week RCT, Langford *et al* found a significant reduction in pain at 10 weeks, however this difference was nonsignificant at study end.^31^ In a 5-week RCT, Rog *et al* found a significant reduction in pain favouring CBM over placebo.^34^

Each of the three studies involving CE administered as capsules showed improvements over placebo. One study measured effects of THC capsules (2.5mg THC) over 12 weeks.^77^ Two studies measured effects of cannabis extract (2.5mg THC/1.25mg CBD) and synthetic THC, both of which showed improvements in pain over placebo for both treatments.^40,42^

Both studies involving whole plant cannabis showed benefit relating to pain reduction. In a cross-sectional study of people living with MS, Clark *et al* reported that 10/12 participants using cannabis for pain management found it to be moderately or completely helpful.^18^ In a 3-day RCT of smoked cannabis (4% THC), Corey-Bloom *et al* (2012) found cannabis to significantly reduce pain scores in people living with MS.^17^

The majority of studies were limited by small numbers of participants, short duration of treatment and some crossover and blinding deficiencies.

***Appendix D - CBM Use for People Living with an Arthritic Condition Experiencing Chronic Pain***

In the RCT,^39^ 58 patients living with rheumatoid arthritis were randomized, double blind to nabiximols (2.7 mg THC/2.5 mg CBD per actuation) nightly or matched placebo for 5 weeks. A mean (SD) of 5.4 (0.84) and 5.3 (1.18) actuations were used in the active and placebo arms respectively. The primary endpoint of reduction of 0-10 numeric rating scale of pain on movement was improved in the active treatment arm [ARR -0.95 (95%CI -1.83, -0.02), P=0.044]. Additionally, statistically and clinically significant improvements in pain at rest, quality of sleep, and the SF-MPQ pain at present component were seen with CBM in comparison to placebo. There was no effect on morning stiffness. Disease activity, as measured by the standard inflammation activity scale, DAS28, was also significantly reduced [ARR -0.76 (95%CI -1.23,-0.28) p=0.002]. Adverse events in the CBM group were all of mild or moderate intensity except for two (6%) rated severe (constipation, malaise) compared with six (22%) in the placebo group. Eight participants (26%) receiving CBM experienced transient dizziness at some point, though in all cases this was rated as mild. No serious adverse events or withdrawals due to adverse events were noted in the active treatment group. The study was limited by the small number of subjects, short treatment duration and modest effect size.

In a study of people living with PTSD^24^, an online survey was completed at baseline, 4-months, and 10-months after initiating use of cannabis. 32 of 588 participants reported living with arthritis at 4-months follow up.^24^ Within this group 68.8% (22/32) reported improvement of arthritis symptoms following medical cannabis use, however this change did not reach statistical significance.

A study, currently published in abstract form,^48^ randomized, double-blind, 320 adult participants with knee osteoarthritis 1:1:1 to CBD 125 mg, 250 mg or placebo applied topically BID for 12 weeks. Randomization followed a 1-week washout period of all anti-inflammatory or analgesic medications other than acetaminophen (paracetamol). Although a numeric benefit was noted, the primary efficacy endpoint of change from baseline in the weekly mean of the 24-hour average worst pain score at week 12, did not reach statistical significance. A key secondary endpoint was a responder analysis, defined as average weekly improvement in worst pain score of > 30% and improvement in WOMAC physical function subscale of at least 20% at last observation which did show a significant benefit for low dose CBD compared to placebo [RRR 52.7% vs 34.1%, p=0.016]. Significant gender differences were noted with the responder analysis and pain scores benefiting men more than women.

The Canadian Rheumatology Association published a position statement on the use of CBM in rheumatic diseases.^49^ The statement, recognizing the paucity of evidence supporting the use CBM, acknowledges that medical cannabis may provide symptom relief for some patients with rheumatic diseases. They emphasize medical cannabis is not an alternative to standard care for any rheumatic disease, and clinicians should adhere to current treatment standards and guidelines for rheumatic disease management.

Other studies that included participants with arthritis found beneficial results in the overall population, but did not report a separate analysis, specific to participants living with arthritis.^3,8,9,20^

***Appendix E - CBM Use for People Living with Fibromyalgia and Chronic Pain***

In an open-label study, two thirds of study participants living with fibromyalgia responded well to sublingual THC treatment.^44^ One of the two remaining studies focused exclusively on patients living with fibromyalgia who have chronic low-back pain.^23^ In this observational cross-over study, Yassin *et al* found cannabis (1:4 THC to CBD, <less than 5% THC) to produce a significant reduction in pain intensity when added to standard analgesic therapy. Participants (N=31; 90% female) were given duloxetine 30mg once daily and oxycodone 5 mg/naloxone 2.5 mg two to three times per day from baseline to three months until stabilized.^23^ Participants were then provided with smoked/vaporized cannabis for the remaining six months (in addition to standard therapy). They were started on 20 grams per month (0.7 grams per day) for three months, with the option of increasing the dose to 30 grams per month (1 gram per day) for the last three months.

VAS pain (0-10; 10 = strongest pain imaginable) ratings did not significantly change from baseline [mean (SD): 8.1(1.2)] to three months post standard analgesic therapy [8.1(1.4)]. However, after cannabis was initiated in addition to standard analgesic therapy, VAS pain ratings significantly improved at three months post-cannabis initiation [5.3 (1.3)] and at six months post-cannabis initiation [3.3 (2.2); both p<0.0001].^23^ Improvements in disability related to spine disorder were also seen from baseline [77.5(10.6)] to three months post-standard therapy [73.7(11.4)] and at three [45.9(19.1)] and six months [30.7(13.6)] post-cannabis initiation (both p<0.0001), on the Oswestry Disability Index (ODI) used to assess functional limitation due to spine disorder. Pain reduction was also reflected in patient self-ratings of overall improvement which changed at six months post-cannabis by a mean baseline difference of 3.3 (95% CI 2.2 to 4.1; SE = 0.31; p<0.0001) as measured by the Patient’s Global Impression of Change (PGIC) scale.^23^ Changes in lumbar flexion, assessed by the Schober test at six months post-cannabis initiation and at three months post-opioid therapy, were each statistically significant from baseline (MD = 1.6; 95% CI 0.4 to 2.8; SE = 0.46, p = 0.0043 and MD = 1.8; 95% CI 0.6 to 3.0; SE = 0.46; p = 0.0010, respectively). Of important note, prescription medications were discontinued or decreased for the majority of patients following cannabis therapy initiation, greatest after six months of cannabis treatment (Student’s t-test 4.5, p<0.001).^23^

Additionally, cannabis was well tolerated; no participants stopped treatment due to adverse events. The most common adverse events were reddening of the eyes (90.3%), increased appetite (16.1%), and sore throat (9.7%) and were mild in nature, not requiring alteration of cannabis doses.^23^ In comparison, the most common adverse events during standardized analgesic therapy included constipation (48.4%), loss of appetite (25.8%), flat affect (16.1%) and hemorrhoids (12.9%) with six participants stopping analgesics due to side effects and one requiring surgery for hemorrhoids.^23^ This study was limited by a lack of a control or placebo group, its small sample size and the sub-population of fibromyalgia patients who also had low-back pain. Additionally, participants were not asked to refrain from other medications and analgesics during the study, potentially confounding the results. Pain ratings were non-responsive to opioid treatment, this may be because access to cannabis was for those with limited pain relief from standard analgesic therapy. Future studies can minimize this bias through the use of a placebo group. Additionally, there was no mention of cannabis education and individualized dosing plans. The lack of this can lead to inadequate pain relief and/or increased adverse effects. Inclusion of a structured cannabis education program and individualised dosing regimens can minimize this gap in medical cannabis intervention.

In an additional study by Habib and Artul,^50^ all 26 participants with fibromyalgia (73% female; mean age = 37.8 ± 7.6 years) receiving medical cannabis (mean dose = 26 ± 8.3 g per month; mean duration = 10.4 ± 11.3 months) from two hospitals in Israel significantly improved on every parameter included on the Revised Fibromyalgia Impact Questionnaire (FIQR). Of note 50% of the study participants ceased other medications used to treat fibromyalgia (Habib & Artul, 2018).^50^ Only 30% of patients experienced very mild side effects.

Fibromyalgia patients are not a homogenous population. Rather, fibromyalgia is a complex condition, frequently occurring with other pain syndromes and symptom clusters. As a result, objective measures of pain, via validated questionnaires are difficult to subcategorize into specific types of pain (ie back pain vs. fibromyalgia pain) in any one individual with fibromyalgia and back pain. Interpretation of these results needs to be considered on a case-by-case basis when extrapolating this evidence to other fibromyalgia patients.

## *Appendix F - CBM Use for People Experiencing Chronic Headache and Migraine*

The only RCT was published in abstract format only. Nicolodi *et al* examined the therapeutic effect of 19% THC and <0.4% THC, 9% CBD in prophylaxis and acute treatment of chronic cluster headache (n=48) and migraine (n=79).^51^ Following a dose-finding study in 48 participants with chronic migraine with THC + CBD, participants with migraine were randomized to 3 months of treatment with amitriptyline 25 mg orally daily or 200 mg THC+CBD/day in 200 ml 50% fat emulsion. Cluster headache participants received 480 mg/day of verapamil as the comparator. Acute treatment consisted of 200 mg THC + CBD or sumatriptan 6 mg subcutaneous injection. THC + CBD and amitriptyline used as migraine prophylaxis both yielded a benefit of approximately 40% pain relief over baseline. THC + CBD used as prophylaxis induced negligible improvement with regards to severity or number of attacks in those with cluster headache. In acute treatment, in both participants with migraine and participants with cluster headache, THC + CBD reduced pain severity by 43% in persons with a history of migraine as children but THC + CBD did not reduce pain severity in those without the childhood history.

The study by Rhyne *et al* was a retrospective chart review conducted at two medical cannabinoid specialty clinics in Colorado that included adults with a primary diagnosis of migraine headache.^52^ Participants used medical cannabis as either migraine treatment or prophylaxis and had to have at least 1 follow-up visit to be included in the study. Although 262 patients were identified, more than half of these individuals were excluded from the analysis due to lack of follow up. A mean reduction in migraine headaches per month from 10.4 to 4.6 was observed in medical cannabis users with 85.1% reporting a decrease in the frequency of migraine headaches. As is common with retrospective chart review, documentation was not consistent across patients. Greater than half of patients used 2 or more forms of cannabis (ie, vaping, edibles, topicals, smoked), with vaping being the most common method. It is not clear whether individuals who did not have at least 1 follow-up visit differed in a systematic way from those who did follow-up, and specifically whether they did not pursue follow-up because the cannabis was not effective in relieving symptoms. Furthermore, as more than half of individuals used 2 or more forms of cannabis, it is unknown whether the form of administration influenced the outcomes.^52^

Chan *et al* examined patient-reported outcomes in patients using medical cannabis for PTSD in Canada.^24^A voluntary online survey was completed by patients at baseline, 4 months and 10 months after starting cannabis from a single medical cannabis provider in Ontario, Canada.^24^ Of those experiencing migraines, 77% reported improvement at 4 months, however, this was not statistically significant. The number of patients approached who declined participation is not mentioned, therefore it is not possible to determine whether participants who refused participation differed from those who agreed to participate. Furthermore, at baseline 86% of participants were already using cannabis which may have resulted in a selection bias.

Woolridge *et al* examined symptom management with cannabis in people living with HIV in a cross-sectional, single clinic study in the United Kingdom.^16^ A 93% response rate was achieved by the 523 questionnaires completed. Of respondents, 27% used cannabis to treat symptoms associated with HIV. Of the 46 participants experiencing headaches, 65% reported improvements with cannabis use.^16^

In summary, there is only 1 RCT that addressed THC + CBD use for migraine or cluster headache, presented in abstract form only. The other three studies consisted of either retrospective, cross-sectional or longitudinal design with significant methodology issues. While observational studies render it difficult to draw conclusions due to the risk of confounding factors, overall cannabis appears to yield some benefit for headaches and migraines.

Adverse events were consistent and commonly reported in included studies. Typically, adverse events were reported as mild to moderate,^5,10,15,34,41^ and often included no serious adverse events.^5,8,13,27,35,38^ The most commonly reported adverse events included dizziness, highness/psychoactive effects, drowsiness, headache, dry mouth, increased appetite and nausea. Other reported adverse events included concentration difficulties, eye redness, balance impairment, vomiting and urinary tract infection. One study reported a treatment-related (nabiximols) suicide attempt.^37^ One study reported falls as an adverse event, also associated with nabiximols.^36^ Severe paranoia was also seen as a serious adverse event.^34^ One study noted that adverse events were less common and less severe than a standard opiate analgesic therapy.^23^

***Appendix G – CBM Use for People Living with Chronic Pain and Nausea***

One case series of Canadian patients using cannabis to treat a range of chronic non-cancer pain conditions found 6/15 participants reported an improvement in nausea attributable to cannabis, an equal number stated no difference in nausea, and none found cannabis use worsened nausea.^9^

A similar case series of Canadian participants with a variety of pain syndromes found 13/30 participants reported moderate to complete relief of nausea using an 11-point numerical rating scale (NRS).^8^ All participants reported the benefits of using cannabis outweighed the side effects, and 95% reported a subjective improvement in function. Participants listed a variety of cannabis products and routes of administration that resulted in benefit.^8^

In a group of men with chronic prostatitis or chronic pelvic pain syndrome, 47% of those completing a standardized survey (either online or in an outpatient clinic setting) rated nausea as slightly or much better due to cannabis use.^25^ There was no indication as to how cannabis was used (inhaled vs. oral), nor the dosing participants used to achieve these results.

A cross-sectional study of patients attending a community dispensary documented use of cannabis for a variety of medical conditions, including chronic pain as a primary benefit in 38% of respondents. Those participants reporting nausea as one of the target conditions rated the mean helpfulness of cannabis as 3.3/5.^21^ Use for this condition did not predict problematic cannabis use, as was found for several other motives for use.

***Appendix H - CBM Use for People with Sleep Problems and Symptoms of Sleep Deprivation Experiencing Chronic Pain***

Of the ten studies exploring whole plant cannabis as a potential treatment for sleep problems in people living with chronic pain, efficacy was consistent. A randomized controlled crossover trial with 21 participants living with chronic neuropathic pain found the highest used dose of inhaled cannabis (9.4% THC) resulted in improved sleep initiation and less periods of wakefulness (statistically significant compared to placebo, p<0.05).^10^ Differences between lower doses (2.5% and 6% THC) and placebo were nonsignificant. Three pre/post studies found cannabis improved sleep quality,^24,25^ and significantly reduced sleep problems.^2^ However, dosing and product selection was not documented, and no control group was established for comparison. Also, the long follow-up intervals could result in recall bias amongst the participants.

The four cross-sectional/survey studies all documented patient-reported benefit for sleep problems associated with pain. In a cross-sectional study of patients living with MS, 17 of the 18 participants (94%) using cannabis for sleep found it to be moderately to completely helpful.^18^ Similarly, a cross-sectional study of patients attending a chronic pain clinic found that 23 of 25 (92%) participants who reported cannabis use for sleep found at least moderate benefit.^6^ Two cross-sectional studies of patients using cannabis for a variety of conditions (MS, chronic pain, HIV-related issues, etc.) reported cannabis to be moderately helpful for improving sleep,^22^ and treating insomnia.^21^ Bonn-Miller *et al* also noted when participants’ cannabis use was targeted for sleep problems, it was associated with less problematic use (e.g., cannabis use disorder).^21^

The two case series studies by Ware *et al*^9^ and Lynch *et al*,^8^ documented cannabis (inhaled or oral routes) improved patient-reported sleep issues in 73% and 78% of their respective samples. These studies were small and did not document the dosing or the composition (THC-rich, CBD-rich or THC/CBD) of the cannabis used.

Cannabis in the form of extracts administered via oromucosal spray (nabiximols) were also found to be consistently beneficial for improving sleep in patients diagnosed with MS. Three separate RCTs found a significant improvement in sleep quality in favour of nabiximols (2.7mg THC + 2.5mg CBD) over placebo.^27,32,33^ These results also carried over from the initial short-term study to long term follow up in two separate studies.^32,36^

A multinational group studied using nabiximols to treat central neuropathic pain and spasticity in MS found difference between treatment and placebo at end of initial parallel group phase (phase A); however, during phase B (randomized post withdrawal from treatment) a significant improvement in sleep quality was noted in favour of those who remained on cannabis treatment.^31^ A British led study of participants with MS using nabiximols for spasticity also produced mixed results.^30^ Their RCT found a trend towards benefit in sleep quality for patients using nabiximols, but this was not statistically significant. A post hoc analysis demonstrated that 61% of participants who experienced >30% reduction in spasticity (labelled “responders”) also experienced improvements in their numerical rating scale (NRS) sleep scores.^30^

In two similar RCTs, Notcutt *et al*^53^ and Wade *et al*^36^ found significant improvements in sleep when participants with a variety of neuropathic pain conditions (majority diagnosed with MS) were given extracts that contained equal amounts of THC (2.5mg) and CBD (2.5mg), delivered sublingually. Notcutt *et al* also found significant improvements using extracts that contained 2.5mg THC or 2.5mg CBD when delivered individually.^53^ This result was not seen in the trial by Wade *et al* despite a similar patient population.^36^

In another crossover RCT with patients who were diagnosed with neuropathic pain due to brachial plexus avulsion, two CE sprays (THC 2.7mg and THC + CBD, 2.7mg + 2.5mg) saw significant improvements in both sleep quality and sleep disturbance over placebo.^39^

One study used nabiximols for treatment of pain due to rheumatoid arthritis (RA), finding a statistically significant improvement in the quality of sleep from baseline by 1.17 points on 11-point NRS (p=0.027).^39^ The authors postulated the improvement in sleep was probably due mainly to nocturnal symptom relief as the dose not high enough for hypnotic effect.^54^

Two of the most recent reports from an international multicentre trial group evaluated the efficacy, safety and tolerability of nabiximols for treatment of peripheral neuropathic pain (having a diagnosis of post-herpetic neuralgia, peripheral neuropathy, focal nerve lesion or CRPS-II).^35,37^ Sleep quality measured at regular intervals over the initial 15 week RCT showed a significant improvement compared to placebo (p=0.007).^35^ An open-label extension trial for a further 38 week period showed those significant sleep improvements achieved during parent RCTs were maintained, supporting the conclusion of long-term efficacy of nabiximols.^37^

Three studies measured the effects of cannabis extract capsules on sleep in participants with MS.^40–42^ All CBM treatments (capsules with THC 2.5mg + CBD 1.25mg,^40,42^ capsules with 2.5mg THC alone,^41^ and synthetic 2.5mg THC [dronabinol])^42^ resulted in significant improvement in sleep quality, in both the short term and longer-term phases. Each of these studies were randomized and placebo-controlled, of sufficient length and showed benefits for pain as well as sleep.^40–42^

Several of these studies documented a variety of adverse events during the trial period related to sleep, including sedation,^35,44^ somnolence,^30–35,37,38,41^ drowsiness,^6,10,39,53^ and sleepiness.^10,24,36^ Sleepiness, drowsiness, fatigue and sedation were also commonly noted as adverse events in studies that did not measure a direct relationship between sleep and CBM use.^14,15,17,44,45,51^

In a randomized, double blind, crossover, placebo controlled trial of 38 people living with neuropathic pain treated with smoked, high (7% THC) and low dose (3.5% THC) cigarettes, hunger, on a 100 mm VAS, increased over time in both treatment groups compared with placebo (*P* <0.001). The difference between the high and low dose groups was not significant (P=.61).^12^ In a series of 20 individual double blind, patient crossover RCTs, Wade *et al* found that whole plant derived THC extract significantly increased appetite, on a 100 mm VAS, compared with placebo [45.6 (SD 26.3) vs 39.0 (SD 25.9) respectively P < 0.05] when delivered sublingually for two weeks.^36^ However, CBD extract and THC+CBD did not significantly differ compared with placebo. A wide, self-titrated, dosage range was used with a mean of 23.5 mg THC daily. Treatment was well tolerated with intoxication noted as the most common side effect. Two similar case series studies found that cannabis improved appetite in 14/30 (47%)^8^, and 9/15 (60%)^9^ chronic pain participants, respectively. Both case series are limited by self-selection of participants as medical cannabis users and no reporting of statistical analysis. A cross-sectional study of 217 people using medical cannabis in California found that 29.7% and 8.8% reported appetite deficiency as a reason or the primary reason for use, respectively.^21^ Cannabis was noted to be moderately helpful on a 5-point Likert scale (3.61, SD 1.26). Although the authors noted that individuals with appetite loss found cannabis to be particularly helpful, compared to other uses, findings also suggest that individuals using cannabis for this purpose may be more prone to problematic use. Woolridge *et al* conducted a questionnaire with 523 people living with HIV in an outpatient clinic.^16^ In this group, over a quarter (n=143) had or were currently using cannabis to treat symptoms related to HIV. Lack of appetite was the most frequent symptom reported with 97% of individuals experiencing improvement with cannabis use [p<0.0001 vs no change]. Smoking was the most common method of administration, being used by 71% of individuals, with 27% of individuals using oral formulations. Once daily was the most common frequency of use at 36%, with 24% of individuals reporting use four or more times daily. The study results are limited by patient self-selection for cannabis use. In a study of persons living with PTSD, Chan *et al* found no significant change in participant appetite following the consumption of medical cannabis for 10 months.^24^ Increased appetite was observed as an expected side effect of cannabis consumption in other included studies.^40^

## *Appendix I - CBM Use for People Living with Chronic Pain Experiencing Appetite Loss*

In a randomized, double blind, crossover, placebo controlled trial of 38 people living with neuropathic pain treated with smoked, high (7% THC) and low dose (3.5% THC) cigarettes, hunger, on a 100 mm VAS, increased over time in both treatment groups compared with placebo (*P* <0.001). The difference between the high and low dose groups was not significant (P=.61).^12^ In a series of 20 individual double blind, patient crossover RCTs, Wade *et al* found that whole plant derived THC extract significantly increased appetite, on a 100 mm VAS, compared with placebo [45.6 (SD 26.3) vs 39.0 (SD 25.9) respectively P < 0.05] when delivered sublingually for two weeks.^36^ However, CBD extract and THC+CBD did not significantly differ compared with placebo. A wide, self-titrated, dosage range was used with a mean of 23.5 mg THC daily. Treatment was well tolerated with intoxication noted as the most common side effect. Two similar case series studies found that cannabis improved appetite in 14/30 (47%)^8^, and 9/15 (60%)^9^ chronic pain participants, respectively. Both case series are limited by self-selection of participants as medical cannabis users and no reporting of statistical analysis. A cross-sectional study of 217 people using medical cannabis in California found that 29.7% and 8.8% reported appetite deficiency as a reason or the primary reason for use, respectively.^21^ Cannabis was noted to be moderately helpful on a 5-point Likert scale (3.61, SD 1.26). Although the authors noted that individuals with appetite loss found cannabis to be particularly helpful, compared to other uses, findings also suggest that individuals using cannabis for this purpose may be more prone to problematic use. Woolridge *et al* conducted a questionnaire with 523 people living with HIV in an outpatient clinic.^16^ In this group, over a quarter (n=143) had or were currently using cannabis to treat symptoms related to HIV. Lack of appetite was the most frequent symptom reported with 97% of individuals experiencing improvement with cannabis use [p<0.0001 vs no change]. Smoking was the most common method of administration, being used by 71% of individuals, with 27% of individuals using oral formulations. Once daily was the most common frequency of use at 36%, with 24% of individuals reporting use four or more times daily. The study results are limited by patient self-selection for cannabis use. In a study of persons living with PTSD, Chan *et al* found no significant change in participant appetite following the consumption of medical cannabis for 10 months.^24^ Increased appetite was observed as an expected side effect of cannabis consumption in other included studies.^40^

## *Appendix J - CBM Use for People with Chronic Pain Experiencing Post-Traumatic Stress Disorder (PTSD)*

Two studies within this review included analysis of cannabis as a treatment for PTSD symptoms.^21,24^ A cross-sectional study of dispensary recruited licensed medical cannabis users found that among the sub-sample of 40 participants reporting PTSD as a primary condition the mean frequency of use was between 2 and 3 times a day, with daily consumption averaging slightly more than 1 gram per day.^21^ The mean helpfulness of cannabis was rated as 3.5/5 on a Likert scale which was classified as within the *moderately helpful* range, and PTSD ranked second among the 10 conditions for which cannabis was reportedly most effective. The approach to quantifying the effect was descriptive and as such no statistical test was performed. Among the larger sample (n=186), higher levels of traumatic intrusions were associated with higher perceived helpfulness of cannabis (*r*  = .22, *p*< .05).^21^

A longitudinal study recruited participants living with PTSD from a licensed cannabis producer to complete online surveys at baseline, 4 month and 10 months.^24^ The interpretability of findings is complicated by high attrition with approximately 75% of baseline respondents not completing the 4-month follow-up. Nonetheless, 78 of the 139 (56%) participants who remained in the study for the four-month follow-up reported *severe pain* at baseline, compared to only 15 who reported *severe pain* (11%) at 4-months (p < 0.01), which suggests that cannabis was perceived to be an effective analgesic among some participants with PTSD. Improvements were also reported in ability to cope with pain and in overall quality of life (QOL), mood, sleep, and concentration.^24^

***Appendix K - CBM Use for People Living with Chronic Pain Experiencing Anxiety***

The highest quality evidence comes from a crossover RCT of smoked cannabis for neuropathic pain, which found that cannabis with 9.4% THC improved anxiety significantly with a medium sized effect over placebo, whereas cannabis with lower THC amounts (2.5% & 6% THC) did not differ from placebo.^10^ Similarly, an open-label single arm trial of cannabis tea found a significant medium sized reduction in anxiety at 12-months compared with baseline.^3^

Two cross-sectional studies also found cannabis to be moderately helpful for treating anxiety.^16,21^ A study of dispensary recruited licensed medical cannabis users found that anxiety was the condition for which cannabis was most commonly reported to be beneficial; among the sub-sample of 131 participants reporting anxiety as a primary condition the mean frequency of use was between 3 and 4 times a day with daily consumption averaging slightly more than 1 gram per day. The mean helpfulness of cannabis was 3.3/5 which was classified as within the *moderately helpful* range.^21^ A cross-sectional study of patients with HIV also identified anxiety as among the most prominent conditions for which CMB was used and reported that 93% of patients using CBM for this purpose reported improvement due to cannabis.^16^ A longitudinal study that recruited people living with PTSD from a licensed cannabis producer reported that 79% of respondents reported improvements in anxiety at 4 month subsequent to initiating CBM.^24^ Finally, a case series found that 11 of a sample of 30 participants with pain registered with the Canadian government’s CBM program reported moderate to complete relief of anxiety from CBM.^8^

In contrast to these generally positive results, one RCT of nabiximols in a sample of patients with MS found no differences in mean changes between treatment groups in measures of anxiety,^27^ and an RCT that compared the acute effects of laboratory administered THC oil reported no change in anxiety relative to placebo among patients with chronic pain.^43^

***Appendix L - CBM Use for People Living with Chronic Pain Experiencing Depression***

Of the 12 studies included in this review reporting on relationships between non-synthetic CBM use and depression in patients with chronic pain, six involved whole plant cannabis either inhaled or ingested,^3,10,16,18,21,24^ and six involved CEs consumed through oromucosal spray or consumed as a capsule.^27,32,33,40,42,53^

The highest quality evidence comes from a crossover RCT of smoked cannabis for neuropathic pain, which found that cannabis with 9.4% THC improved depressive symptoms significantly with a medium sized effect over placebo, whereas lower THC cannabis (2.5% & 6% THC) did not differ from placebo.^10^ Three cross-sectional studies also report antidepressant effects of CBM. A study of patients with HIV identified depression as among the most prominent conditions for which CBM was used and reported that 86% of participants using CBM for this purpose reported improvement due to cannabis.^16^ Another cross-sectional study reported that all 16 participants living with MS using cannabis for mood found it to be moderately to completely helpful.^18^ A study of licensed medical cannabis users recruited from a dispensary found that depression was among the conditions for which cannabis was most commonly reported to be beneficial; among the sub-sample of 95 participants reporting depression as a primary condition the mean frequency of use was between 3 and 4 times a day with daily consumption averaging slightly more than 1 gram per day. The mean helpfulness of cannabis was rated as 3.29/5 on a Likert scale which was classified as within the *moderately helpful* range.^21^ A non-randomized single arm study also found a significant reduction in depression scores at 12 months in participants with chronic pain treated with cannabis prepared as a tea.^3^ A longitudinal study that recruited participants with PTSD from a licensed cannabis producer indicated that 78% of respondents reported improvements in depression at 4 month subsequent to initiating CBM.^24^

Studies involving the use of CEs are generally less positive regarding the benefits of CBM for treating depression in people with chronic pain. One series of “*N of 1”* studies that began as an open label trial before transitioning to an RCT of different CBM extract delivered through oromucosal spray reported changes in depression among 14/24 participants, with 10 participants changing from *moderate* to either *mild* or *minimal* levels of depression, 3 moving from *severe* to *mild* or *minimal* and one change from *minimal* to *mild*.^53^ In contrast to these positive results, three RCTs found no significant difference between nabiximols and placebo in depression scores.^27,32,33^ Similarly, two studies using CEs capsules reported no significant difference in depression scores between treatment groups and placebos.^40,42^

## *Appendix M - Adjunctive CBM Use for People Living with Chronic Pain Experiencing Unsatisfactory Analgesia from Opioid Treatment*

In a two-arm study with participants with chronic pain (N = 21; 48% female) on a sustained-release opioid regimen [morphine (mean dose 62 mg, taken twice daily) or oxycodone (mean dose 53 mg, twice a day)], pain was significantly decreased after the addition of vaporized cannabis for five days (once a day on days one and five; three times a day for days two to four).^26^ Participants’ pain types included musculoskeletal (17.3%), posttraumatic (19.1%), arthritic (9.5%), peripheral neuropathy (9.5%), cancer (4.8%), fibromyalgia (4.8%), migraine (4.8%), MS (4.8%), sickle cell disease (4.8%) and thoracic outlet syndrome (4.8%). Pain ratings on a VAS (0-100) decreased significantly by an average of 27% [95% confidence interval (CI) 9, 46] with the addition of vaporized cannabis when morphine and oxycodone cohorts were combined, while not producing any significant plasma opioid alterations. Additionally, vaporized cannabis did not produce any significant effects on oxycodone-only kinetics or metabolite outcomes. Cannabis use also did not produce any clinically significant adverse effects and produced minimal subjective effects including a “high” that was not observed with opioids only. The authors concluded that vaporized cannabis augments the analgesic effects of opioids without significantly altering plasma opioid levels.

Similarly, in an observational study among people living with fibromyalgia and low back pain (N=31; 90% female), Yassin *et al* found minimal improvement in pain scores from baseline while on standard analgesic therapy (oxycodone 5 mg/naloxone 2.5 mg twice a day and duloxetine 30 mg once a day).^23^ The addition of cannabis treatment to standard analgesic therapy produced significant improvements in all self-reported outcomes (including: pain intensity, disability, belief of the efficacy of treatment) at three- and six-months post-cannabis initiation (all p’s<0.0001). Range of motion, as assessed by lumbar flexion, improved continuously from month three to month six (p<0.005) post-cannabis initiation and from standard therapy to six months post-initiation of cannabis (p<0.0001). Medication consumption decreased or was discontinued among patients post-cannabis initiation, which was significantly greater at six months compared to when participants were receiving standard therapy only (Student’s t-test 4.5, p<0.001).^23^ Cannabis was well tolerated, adverse effects were minimal and less severe than those related to opioid treatment, requiring no participants to stop treatment or to alter doses. Cannabis-related adverse effects included reddening of the eyes, increased appetite and sore throat. In comparison, opioid-related effects included constipation, loss of appetite, feeling of reduced mental acuity and flat affect and hemorrhoids. In addition, 19.4% of participants stopped treatment and 3.2% were to undergo operation related to hemorrhoids.^23^

A five-day crossover RCT with people living with HIV-associated distal sensory predominant polyneuropathy (DSPN) (N=34 eligible, 28 completers; 0% female) found individually titrated smoked cannabis (1-8% THC) significantly reduced pain compared with placebo.^15^ The majority of included individuals were prescribed combination antiretroviral therapy (93%), had been exposed to potentially neurotoxic dideoxynucleoside reverse transcriptase inhibitors (72%) and had previous exposure to cannabis (96%).^15^ All subjects continued to maintain their regular dosing of concomitant analgesics including opioids, nonsteroidal anti-inflammatory drugs (NSAIDs) and adjunctive pain medications throughout the study. Participants titrated either smoked cannabis or placebo for five consecutive days (4x/day) during the two treatment periods which were separated by a two-week washout period until pain relief without serious and/or intolerable adverse effects was achieved. Pain intensity, as measured by the Descriptor Differential Scale, was significantly decreased with cannabis versus placebo (median difference in DDS pain intensity, 3.3 points, effect size=0.6; p=0.016) with a greater proportion of participants achieving at least 30% reduction in pain for cannabis at 0.46 (95% CI 0.28, 0.65) compared to placebo at 0.18 (95% CI 0.03, 0.32).^15^These results were consistent with VAS ratings (p<0.001), the ITT analysis (p=0.020) and when analyzing the first week of treatment only (p=0.029), with no evidence of sequence effects. To achieve 30% reduction in pain, the NNT was 3.5 (95% CI 1.9, 20.8). Moreover, significant improvements in quality of life, physical disability and mood disturbance were observed among participants, regardless of treatment order, but not between cannabis and placebo groups. Unlike the previous two studies, *Ellis et al* did not observe either a positive or negative interaction between opioids and cannabis.^15^ Adverse effects related to cannabis were considered mild, although greater compared to placebo and included: fatigue, sedation, impairments in concentration, and reduced salivation. Heart rate increases of 30 points or more were more frequent with cannabis (46%) compared to placebo (4%) within 30 minutes of smoking. Subjects withdrew due to intractable, smoking-related coughing symptoms which resolved upon cessation of smoking (3.6%) and acute, cannabis-induced psychosis (3.6%).

A cross-sectional study of people living with chronic non-cancer pain using opioid medications (N = 1514; 44% female) found that participants reported a 50% reduction in pain relief from their opioid medications. In the cannabis for relief subgroup (16% of participants, N=237), the participants, reported a 70% average reduction in the amount of subjective pain relief.This may suggest a potentially compounded analgesic effect of concurrent opioid and cannabis use.^19^ Additionally, 25% of all participants (N=363) stated they would take cannabis for pain relief if it was accessible to them.^19^ Greater pain interference, after controlling for severity, was observed among those using cannabis for pain relief, with users reporting increased relief when combined with opioids compared to the use of opioids solely. Although there were no differences in median prescribed opioid doses or non-adherent use of these doses. Those using cannabis for pain relief were more likely to be male, younger (median 48 years vs. 59 years), have greater pain interference, poorer coping of pain, greater pain severity and were significantly more likely to meet criteria for moderate or severe depression, generalized anxiety disorder.

Limitations of the four included studies were largely due to no control/placebo and/or comparison groups,^23,26^ in addition to small sample sizes.^15,23,26^ Future studies should include placebo groups in order to minimize biases such as ‘placebo-effects’ and other biases specific to studies. For instance, access to cannabis treatment within the study conducted by *Yassin et al*  was contingent inadequate pain relief from standard analgesic therapy, as assessed through pain ratings.^23^ Additionally, *Ellis et al* had potential blinding issues, as participants were able to detect treatment from placebo.^15^ The authors concluded that although a placebo effect was detected, the treatment effects were in addition to the placebo effects.^15^ *Degenhardt et al* were limited by the cross-sectional design of their study which relied on self-reported data and did not assess the amount of cannabis consumed, only the frequency of use.^19^ Studies also did not recruit a representative sample of individuals prescribed opioids for chronic pain.^15,19,23,26^

Several additional studies found improvements in chronic pain associated with CBM use within samples that included participants concurrently using opioids to treat pain.^4,10,20,31,34^ The adjunctive use of medical cannabis with opioids has been shown to be effective in clinical practice for neuropathic and other chronic pain, MS, arthritis, cancer, HIV, anxiety disorders, PTSD, epilepsy and irritable bowel syndrome.^55^ The benefits of medical cannabis are also observed among reductions in underlying symptoms of conditions such as pain, spasms, symptoms of anxiety, seizures, insomnia and nausea.^55^ Among a cross-sectional study of participants with chronic pain (N = 890; 57% response rate) at two centres in Israel, 53.3% were treated with prescription opioids only, 37.0% were treated with medical cannabis only, and the remaining 8.7% were treated with prescription opioids and medical cannabis.^56^ Significant improvements were observed in secondary outcomes such as depression and anxiety for the medical cannabis group compared to the opioid-only and opioid plus cannabis groups (both P<0.01), suggesting that levels of depression and anxiety may be more prevalent among patients receiving opioids only.

### Summary:

The adjunctive use of medical cannabis with prescription opioids for pain management appears to be effective in reducing pain among various patients with chronic neuropathic pain and in improving associated secondary outcomes such as depression, anxiety, and quality of life. All of the four included studies noted a positive effect of adjunctive cannabis among pain outcomes. This adjunctive approach would allow for lower doses of opioids to be used with the addition of cannabis as well as fewer side effects. Adjunctive cannabis appears to be safe and tolerable as no alteration of plasma opioid levels have been observed. Adverse effects related to the adjunctive use of cannabis appear to be minimal and typically mild in nature (fatigue, reddening of the eyes, impairment in concentration), especially compared to side effects produced by opioids Future research is needed to determine whether patients with higher pain scores respond better to cannabis as an adjunct than those with less pain, as was observed among some of the included studies in addition to increasing sample sizes. More research is also required regarding if cannabis should be considered as a treatment option prior to opioid initiation based on the lower risk of adverse effects compared to opioids.

***Appendix N - CBM use and Opioid Sparing for People Using Opioids as Treatment for Chronic Pain***

A cohort study by Vigil *et al* (2017) found positive associations between medical cannabis use and opioid sparing in an American chronic pain sample.^5^ In this study, 37 participants of a medical cannabis program were found to be more likely to cease opioid prescription use (OR 17.27, CI 1.89-157.36, p=0.012), and reduce mean daily opioid consumption (OR 5.12, CI 1.56-16.88, p=0.007), than the comparison group.^5^ Survey results also indicated significant (p<0.001) improvements in pain reduction, QoL, activity levels and concentration for the cannabis group one year after initiation.^5^ The authors noted that cannabis use enabled participants to engage in harm reduction despite a safety comparison of cannabis and opioids being outside the scope of this study.^5^

A prospective open-label trial of medical cannabis found a significant reduction (44%; p<0.001) in opioid consumption at follow-up among participants over six months, which was also accompanied by significant increases in functional outcomes and decreases in pain.^2^ Participants (N = 176; 206 ITT; 48% female) had the right to choose between smoked (cannabis cigarettes), edible (baked cookies), extracts (oil drops) or combined cannabis varieties (for a total dose of 20 gram per month). Smoking was the preferred route (77.3%) followed by extracts (9.7%), edibles (5.1%), smoking plus extracts (4.6%) and extracts plus edibles (3.4%) with a mean (SD) intake (among any route) of 43.2(17.9) grams per month. They were instructed to titrate their dose up to a frequency of three times per day. The cannabinoid concentration in smoked (THC 6-14%; CBD 0.2-3.8%) versus oral (THC 11-19%; CBD 0.5-5.5%) cannabis varied. Main diagnoses included musculoskeletal widespread pain (30.1%), peripheral neuropathic pain (23.8%) and radicular low back pain (18.9%); 93.2% of diagnoses consisting of chronic noncancer pain. Forty-one percent of participants had a median oral morphine equivalent dose of 60 mg (95% CI, 45.0-90.0). Of those still taking opioids at follow-up, the median oral morphine equivalent dose decreased from 60 mg to 45 mg (95% CI, 30.0-90.0) although not statistically significant (p=0.19). Significant improvements were also noted among 65.9% of participants’ pain symptoms, as assessed by the STOPS (Treatment Outcomes in Pain Survey - Short Form) questionnaire from baseline (83.3, 95% CI 79.2-87.5) to 6-months post-treatment (75, 95% CI 70.8-79.2; p<0.001) which remained similar in the PP analysis (p<0.001). Additionally, significant improvements were noted among The Brief Pain Inventory subscales of pain severity and interference (both p<0.01) as well as measures of sleep (p<0.001). Adverse effects resulting from the addition of cannabis to opioids were mild to moderate (5.3%) with 5.1% of participants discontinuing cannabis due to sedation, heaviness, nervousness and concentration difficulties. Serious adverse effects were only experienced among 1.1% of participants, including elevated liver transaminases and confusion requiring hospitalization.

Similarly, Bellnier *et al* found that opioid consumption was reduced by approximately 75% from baseline to three months in a retrospective mirror-image study in participants with chronic pain (N = 29; 65% female) using medical cannabis. Other benefits were cost savings, improvement in quality of life, reduction of opioid-related adverse events, and reduction in pain (including paroxysmal, surface, deep and unpleasant).^1^ The study dose was 10 mg THC/10 mg CBD capsules every 8-12 hours. However, if breakthrough pain was experienced, participants were provided with a vaporizer pen (2mg THC/0.1mg CBD per inhalation). Pain conditions included spinal tissue damage (70%), neuropathies (10%), cancer (10%), irritable bowel disease (7%) and Parkinson disease (3%); of which 90% was chronic noncancer pain. Morphine equivalent reduced from a starting dose of 79.9 mg (0-450mg) to 19.6 mg (range of 0-150; p<0.05) and monthly analgesic costs from $US 354.70 ($0-1838) to $US 241.10 (range $0-477; p<0.05). Almost the entire sample (89.7%) discontinued opioids after three months. Moreover, pain quality, as measured by the Pain Quality Assessment Scale (PQAS), improved from baseline to the end of three months post-cannabis (p<0.0001). After three months of cannabis treatment, ratings for paroxysmal pain improved from 6.76 to 2.04 and deep pain from 5.87 to 2.03 (both p<0.0001) with ratings for unpleasant pain also decreasing from “miserable” to “annoying”. Quality of life ratings also improved from baseline to after three months of cannabis treatment (p<0.0001). Adverse effects were mild in nature and were present in 10% of the sample which differed from what was experienced at the 1-month follow-up (dry mouth, 21%; dizziness, 3%; increased appetite, 3%) to the 3-month follow up (only dry mouth, 10%), indicating a development of tolerance and resolution of adverse events.

Campbell *et al* found that cannabis sometimes or regularly reduced opioid medications among 28% of chronic noncancer pain participants (N = 1514; 66% female).^4^ Participants had pain ranging from back or neck pain (77%), arthritis (62%) and multifactorial pain etiology, and taking a median oral morphine equivalent dose of 75 mg per day (range of 36-150mg per day).^4^ No differences in age, sex, oral morphine equivalent, pain severity or interference emerged between these two groups that experienced an effect of cannabis on opioid consumption compared to those that did not. When bivariate analyses were performed based on frequency of use, those who used cannabis less frequently (<20 days per month) were significantly less likely to discontinue opioids compared to those that didn’t use cannabis at all (RR 0.38; 95% CI 0.17-0.83; p = 0.016). This trend was not observed when comparing those using cannabis more frequently (daily/near daily) to those that did not use cannabis at all (RR 1.05; 95% CI 0.60-1.84; p = 0.85). After a four-year follow-up, those who used cannabis had greater pain severity scores (daily/near-daily use: RR = 1.17, 95% CI 1.03-1.32; less frequent use: RR = 1.14, 95% CI 1.01-1.29), pain interference ratings (daily/near daily use: RR = 1.13, 95% CI 1.03-1.26; less frequent use: RR = 1.21, 95% CI 1.09=1.35), reduced pain self-efficacy ratings (daily/near daily use: RR = 0.98, 95% CI 0.96-1.00; less frequent use: 0.97, 95% CI 0.96-1.00) and greater generalized anxiety severity scores (daily/near daily use: RR = 1.10, 95% CI 1.06-1.15; less frequent use: RR = 1.07, 95% CI 1.03-1.12) compared to participants that did not. Based on this data, the authors acknowledged that the participants included in this study likely consumed cannabis without the guidance of a medical practitioner, and greater reduction in opioids may have been captured with more regular cannabis use.

Two studies reported that the majority of participants reduced their routine pain medications by 60-70%, however the extent to which opioid medications were specifically reduced was unclear.^6,8^ For instance, results from a cross-sectional survey indicated that among 32 participants living with chronic noncancer pain using cannabis as an analgesic, 59% stated the use of cannabis allowed them to reduce their total number of pain medication doses.^6^ In this same study, frequency of use varied from greater than once per day (22%), daily (16%), weekly (25%) and rarely (28%) with smoking cannabis cigarettes as the most common route of administration (81%). Of those using cannabis, 78% of individuals reported moderate pain relief, 92% experienced improvements in sleep, and 96% in mood. Similarly, among a case series investigating participants with chronic treatment-resistant pain (N = 30; 40% female) taking medical cannabis for one to five years (<1-5 g per day; average dose 2.5g per day), all participants reported smoking cannabis some of the time, whereas the oral route was less common.^8^ Lynch *et al* reported that 65% of participants (n=23) decreased other medications including NSAIDs, opioids and antidepressants; which subsequently decreased adverse events. Participants additionally noted improvements in sleep (95.7%), anxiety (47.8%) and mood difficulties (34.8%).^8^ An additional study also found that participants taking long-term opioid therapy (N = 371) and using medical cannabis, reported fewer side effects than those participants who did not take cannabis (median score = 88.0 vs. 92.0, p = 0.04).^20^ However, medical cannabis users did not significantly differ from non-users regarding current prescription opioid dose, pain intensity scores, pain-related disability scores, depression, anxiety or quality of life. Similar mild adverse effects were reported in approximately 75% of participants in both studies; the most common effects reported included, feeling ‘high’, dry mouth, drowsiness, increased appetite and slowed thoughts while pain, sleep and mood improved most frequently with use.^6,8^

Limitations of these seven studies included study designs,^1,2,4–6,8,20^no prior sample size calculationperformed,^2^ unrepresentative samples,^1,2,4,5,20^ small sample sizes,^1,5,6,8^ selection bias,^1,5,6,8^ lack of control group,^2,8^ lack of blinding,^1,5^ absence of baseline measurements,^8^ 3-fold variability in THC concentrations provided,^2^ and a lack opioid sparing reporting.^4,6,8^ Additionally, there were no mention of cannabis education and individualized dosing plans. The lack of these elements can lead to inadequate pain relief and/or increased adverse effects. Inclusion of a structured cannabis education program and individualised dosing regimens can minimize this gap in medical cannabis intervention.

### Summary:

The use of medical cannabis among people with chronic neuropathic pain taking prescription opioids may reduce the reliance on opioid medications leading to a reduction in opioid dose or to a complete opioid taper. The synergy between cannabinoids and opioids can lead to greater beneficial effects while reducing the adverse events associated with opioids, especially among high doses of opioids. Patients receiving high doses of opioids for pain management may notice greater opioid sparing effects. Associated secondary benefits such as analgesic cost savings and improved functional outcomes are also observed with the addition of medical cannabis. Patients using cannabinoids for the purpose of opioid sparing should follow a structured approach (Figure 3), under clinical guidance and with regular monitoring. Cannabinoid use may be considered for opioid sparing when patients are not reaching pain goals, are experiencing opioid-related adverse events, are experiencing risk factors for opioid-related harms, or when opioid doses exceed 90 MEQ. Initiation of CBD-dominant treatment should be titrated to a maximum of 30-40mg THC/day. A slow taper of opioid use (5-10% morphine equivalent dose (MED)) should be sought until pain/adverse event objectives are achieved (Figure 3). Future research should focus on opioid sparing as a primary outcome and include large, representative samples to further clarify the relationship between cannabis and resulting reduced opioid doses.

***References***

1. Bellnier T, Brown GW, Ortega TR. Preliminary evaluation of the efficacy, safety, and costs associated with the treatment of chronic pain with medical cannabis. *Ment Health Clin*. 2018;8(3):110-115. doi:10.9740/mhc.2018.05.110

2. Haroutounian S, Ratz Y, Ginosar Y, et al. The Effect of Medicinal Cannabis on Pain and Quality-of-Life Outcomes in Chronic Pain: A Prospective Open-label Study. *Clin J Pain*. 2016;32(12):1036-1043. doi:10.1097/AJP.0000000000000364

3. Poli P, Crestani F, Salvadori C, Valenti I, Sannino C. Medical Cannabis in Patients with Chronic Pain: Effect on Pain Relief, Pain Disability, and Psychological aspects. A Prospective Non randomized Single Arm Clinical Trial. *Clin Ter*. 2018;169(3):e102-e107. doi:10.7417/T.2018.2062

4. Campbell G, Hall WD, Peacock A, et al. Effect of cannabis use in people with chronic non-cancer pain prescribed opioids: findings from a 4-year prospective cohort study. *Lancet Public Health*. 2018;3(7):e341-e350. doi:10.1016/S2468-2667(18)30110-5

5. Vigil JM, Stith SS, Adams IM, Reeve AP. Associations between medical cannabis and prescription opioid use in chronic pain patients: A preliminary cohort study. *PLoS One*. 2017;12(11):e0187795. doi:10.1371/journal.pone.0187795

6. Ware MA, Doyle CR, Woods R, Lynch ME, Clark AJ. Cannabis use for chronic non-cancer pain: results of a prospective survey. *Pain*. 2003;102(1-2):211-216. doi:10.1016/s0304-3959(02)00400-1

7. Fanelli G, De Carolis G, Leonardi C, et al. Cannabis and intractable chronic pain: an explorative retrospective analysis of Italian cohort of 614 patients. *J Pain Res*. 2017;10:1217-1224. doi:10.2147/JPR.S132814

8. Lynch ME, Young J, Clark AJ. A case series of patients using medicinal marihuana for management of chronic pain under the Canadian Marihuana Medical Access Regulations. *J Pain Symptom Manage*. 2006;32(5):497-501. doi:10.1016/j.jpainsymman.2006.05.016

9. Ware MA, Gamsa A, Persson J, Fitzcharles MA. Cannabis for chronic pain: case series and implications for clinicians. *Pain Res Manag*. 2002;7(2):95-99. doi:10.1155/2002/380509

10. Ware MA, Wang T, Shapiro S, et al. Smoked cannabis for chronic neuropathic pain: a randomized controlled trial. *CMAJ*. 2010;182(14):E694-701. doi:10.1503/cmaj.091414

11. Wilsey B, Marcotte T, Deutsch R, Gouaux B, Sakai S, Donaghe H. Low-dose vaporized cannabis significantly improves neuropathic pain. *J Pain*. 2013;14(2):136-148. doi:10.1016/j.jpain.2012.10.009

12. Wilsey B, Marcotte T, Tsodikov A, et al. A randomized, placebo-controlled, crossover trial of cannabis cigarettes in neuropathic pain. *J Pain*. 2008;9(6):506-521. doi:10.1016/j.jpain.2007.12.010

13. Wilsey B, Marcotte TD, Deutsch R, Zhao H, Prasad H, Phan A. An Exploratory Human Laboratory Experiment Evaluating Vaporized Cannabis in the Treatment of Neuropathic Pain From Spinal Cord Injury and Disease. *J Pain*. 2016;17(9):982-1000. doi:10.1016/j.jpain.2016.05.010

14. Abrams DI, Jay CA, Shade SB, et al. Cannabis in painful HIV-associated sensory neuropathy: a randomized placebo-controlled trial. *Neurology*. 2007;68(7):515-521. doi:10.1212/01.wnl.0000253187.66183.9c

15. Ellis RJ, Toperoff W, Vaida F, et al. Smoked medicinal cannabis for neuropathic pain in HIV: a randomized, crossover clinical trial. *Neuropsychopharmacology*. 2009;34(3):672-680. doi:10.1038/npp.2008.120

16. Woolridge E, Barton S, Samuel J, Osorio J, Dougherty A, Holdcroft A. Cannabis use in HIV for pain and other medical symptoms. *J Pain Symptom Manage*. 2005;29(4):358-367. doi:10.1016/j.jpainsymman.2004.07.011

17. Corey-Bloom J, Wolfson T, Gamst A, et al. Smoked cannabis for spasticity in multiple sclerosis: a randomized, placebo-controlled trial. *CMAJ*. 2012;184(10):1143-1150. doi:10.1503/cmaj.110837

18. Clark AJ, Ware MA, Yazer E, Murray TJ, Lynch ME. Patterns of cannabis use among patients with multiple sclerosis. *Neurology*. 2004;62(11):2098-2100. doi:10.1212/01.wnl.0000127707.07621.72

19. Degenhardt L, Lintzeris N, Campbell G, et al. Experience of adjunctive cannabis use for chronic non-cancer pain: findings from the Pain and Opioids IN Treatment (POINT) study. *Drug Alcohol Depend*. 2015;147:144-150. doi:10.1016/j.drugalcdep.2014.11.031

20. Nugent SM, Yarborough BJ, Smith NX, et al. Patterns and correlates of medical cannabis use for pain among patients prescribed long-term opioid therapy. *Gen Hosp Psychiatry*. 2018;50:104-110. doi:10.1016/j.genhosppsych.2017.11.001

21. Bonn-Miller MO, Boden MT, Bucossi MM, Babson KA. Self-reported cannabis use characteristics, patterns and helpfulness among medical cannabis users. *Am J Drug Alcohol Abuse*. 2014;40(1):23-30. doi:10.3109/00952990.2013.821477

22. Brunt TM, van Genugten M, Höner-Snoeken K, van de Velde MJ, Niesink RJM. Therapeutic satisfaction and subjective effects of different strains of pharmaceutical-grade cannabis. *J Clin Psychopharmacol*. 2014;34(3):344-349. doi:10.1097/JCP.0000000000000129

23. Yassin M, Oron A, Robinson D. Effect of adding medical cannabis to analgesic treatment in patients with low back pain related to fibromyalgia: an observational cross-over single centre study. *Clin Exp Rheumatol*. 2019;37 Suppl 116(1):13-20.

24. Chan S, Blake A, Wolt A, et al. Medical cannabis use for patients with post-traumatic stress disorder (PTSD). *J Pain Manage*. 2017;10(4):385-396.

25. Tripp DA, Nickel JC, Katz L, Krsmanovic A, Ware MA, Santor D. A survey of cannabis (marijuana) use and self-reported benefit in men with chronic prostatitis/chronic pelvic pain syndrome. *Can Urol Assoc J*. 2014;8(11-12):E901-905. doi:10.5489/cuaj.2268

26. Abrams DI, Couey P, Shade SB, Kelly ME, Benowitz NL. Cannabinoid-opioid interaction in chronic pain. *Clin Pharmacol Ther*. 2011;90(6):844-851. doi:10.1038/clpt.2011.188

27. Rog DJ, Nurmikko TJ, Friede T, Young CA. Randomized, controlled trial of cannabis-based medicine in central pain in multiple sclerosis. *Neurology*. 2005;65(6):812-819. doi:10.1212/01.wnl.0000176753.45410.8b

28. Rog DJ, Nurmikko TJ, Young CA. Oromucosal delta9-tetrahydrocannabinol/cannabidiol for neuropathic pain associated with multiple sclerosis: an uncontrolled, open-label, 2-year extension trial. *Clin Ther*. 2007;29(9):2068-2079. doi:10.1016/j.clinthera.2007.09.013

29. Russo M, Naro A, Leo A, et al. Evaluating Sativex® in Neuropathic Pain Management: A Clinical and Neurophysiological Assessment in Multiple Sclerosis. *Pain Med*. 2016;17(6):1145-1154. doi:10.1093/pm/pnv080

30. Collin C, Ehler E, Waberzinek G, et al. A double-blind, randomized, placebo-controlled, parallel-group study of Sativex, in subjects with symptoms of spasticity due to multiple sclerosis. *Neurol Res*. 2010;32(5):451-459. doi:10.1179/016164109X12590518685660

31. Langford RM, Mares J, Novotna A, et al. A double-blind, randomized, placebo-controlled, parallel-group study of THC/CBD oromucosal spray in combination with the existing treatment regimen, in the relief of central neuropathic pain in patients with multiple sclerosis. *J Neurol*. 2013;260(4):984-997. doi:10.1007/s00415-012-6739-4

32. Novotna A, Mares J, Ratcliffe S, et al. A randomized, double-blind, placebo-controlled, parallel-group, enriched-design study of nabiximols* (Sativex(®) ), as add-on therapy, in subjects with refractory spasticity caused by multiple sclerosis. *Eur J Neurol*. 2011;18(9):1122-1131. doi:10.1111/j.1468-1331.2010.03328.x

33. Wade DT, Makela P, Robson P, House H, Bateman C. Do cannabis-based medicinal extracts have general or specific effects on symptoms in multiple sclerosis? A double-blind, randomized, placebo-controlled study on 160 patients. *Mult Scler*. 2004;10(4):434-441. doi:10.1191/1352458504ms1082oa

34. Nurmikko TJ, Serpell MG, Hoggart B, Toomey PJ, Morlion BJ, Haines D. Sativex successfully treats neuropathic pain characterised by allodynia: a randomised, double-blind, placebo-controlled clinical trial. *Pain*. 2007;133(1-3):210-220. doi:10.1016/j.pain.2007.08.028

35. Serpell M, Ratcliffe S, Hovorka J, et al. A double-blind, randomized, placebo-controlled, parallel group study of THC/CBD spray in peripheral neuropathic pain treatment. *Eur J Pain*. 2014;18(7):999-1012. doi:10.1002/j.1532-2149.2013.00445.x

36. Wade DT, Robson P, House H, Makela P, Aram J. A preliminary controlled study to determine whether whole-plant cannabis extracts can improve intractable neurogenic symptoms. *Clin Rehabil*. 2003;17(1):21-29. doi:10.1191/0269215503cr581oa

37. Hoggart B, Ratcliffe S, Ehler E, et al. A multicentre, open-label, follow-on study to assess the long-term maintenance of effect, tolerance and safety of THC/CBD oromucosal spray in the management of neuropathic pain. *J Neurol*. 2015;262(1):27-40. doi:10.1007/s00415-014-7502-9

38. Berman JS, Symonds C, Birch R. Efficacy of two cannabis based medicinal extracts for relief of central neuropathic pain from brachial plexus avulsion: results of a randomised controlled trial. *Pain*. 2004;112(3):299-306. doi:10.1016/j.pain.2004.09.013

39. Blake DR, Robson P, Ho M, Jubb RW, McCabe CS. Preliminary assessment of the efficacy, tolerability and safety of a cannabis-based medicine (Sativex) in the treatment of pain caused by rheumatoid arthritis. *Rheumatology (Oxford)*. 2006;45(1):50-52. doi:10.1093/rheumatology/kei183

40. Zajicek J, Fox P, Sanders H, et al. Cannabinoids for treatment of spasticity and other symptoms related to multiple sclerosis (CAMS study): multicentre randomised placebo-controlled trial. *Lancet*. 2003;362(9395):1517-1526. doi:10.1016/S0140-6736(03)14738-1

41. Zajicek JP, Hobart JC, Slade A, Barnes D, Mattison PG, MUSEC Research Group. Multiple sclerosis and extract of cannabis: results of the MUSEC trial. *J Neurol Neurosurg Psychiatry*. 2012;83(11):1125-1132. doi:10.1136/jnnp-2012-302468

42. Zajicek JP, Sanders HP, Wright DE, et al. Cannabinoids in multiple sclerosis (CAMS) study: safety and efficacy data for 12 months follow up. *J Neurol Neurosurg Psychiatry*. 2005;76(12):1664-1669. doi:10.1136/jnnp.2005.070136

43. Weizman L, Dayan L, Brill S, et al. Cannabis analgesia in chronic neuropathic pain is associated with altered brain connectivity. *Neurology*. 2018;91(14):e1285-e1294. doi:10.1212/WNL.0000000000006293

44. Haroutiunian S, Rosen G, Shouval R, Davidson E. Open-label, add-on study of tetrahydrocannabinol for chronic nonmalignant pain. *J Pain Palliat Care Pharmacother*. 2008;22(3):213-217. doi:10.1080/15360280802251215

45. Cuñetti L, Manzo L, Peyraube R, Arnaiz J, Curi L, Orihuela S. Chronic Pain Treatment With Cannabidiol in Kidney Transplant Patients in Uruguay. *Transplant Proc*. 2018;50(2):461-464. doi:10.1016/j.transproceed.2017.12.042

46. Moulin D, Boulanger A, Clark AJ, et al. Pharmacological management of chronic neuropathic pain: revised consensus statement from the Canadian Pain Society. *Pain Res Manag*. 2014;19(6):328-335. doi:10.1155/2014/754693

47. Russo EB, Guy GW, Robson PJ. Cannabis, pain, and sleep: lessons from therapeutic clinical trials of Sativex, a cannabis-based medicine. *Chem Biodivers*. 2007;4(8):1729-1743. doi:10.1002/cbdv.200790150

48. Hunter D, Oldfield G, Tich N, Messenheimer J, Sebree T. Synthetic transdermal cannabidiol for the treatment of knee pain due to osteoarthritis. *Osteoarthritis and Cartilage*. 2018;26:S26. doi:10.1016/j.joca.2018.02.067

49. Fitzcharles MA, Niaki OZ, Hauser W, Hazlewood G, Canadian Rheumatology Association. Position Statement: A Pragmatic Approach for Medical Cannabis and Patients with Rheumatic Diseases. *J Rheumatol*. 2019;46(5):532-538. doi:10.3899/jrheum.181120

50. Habib G, Artul S. Medical Cannabis for the Treatment of Fibromyalgia. *J Clin Rheumatol*. 2018;24(5):255-258. doi:10.1097/RHU.0000000000000702

51. Nicolodi M, Sandoval V, Terrine A. Therapeutic use of cannabinoids - dose finding, effects, and pilot data of effects in chronic migraine and cluster headache. In: ; 2017.

52. Rhyne DN, Anderson SL, Gedde M, Borgelt LM. Effects of Medical Marijuana on Migraine Headache Frequency in an Adult Population. *Pharmacotherapy*. 2016;36(5):505-510. doi:10.1002/phar.1673

53. Notcutt W, Price M, Miller R, et al. Initial experiences with medicinal extracts of cannabis for chronic pain: results from 34 “N of 1” studies. *Anaesthesia*. 2004;59(5):440-452. doi:10.1111/j.1365-2044.2004.03674.x

54. Nicholson AN, Turner C, Stone BM, Robson PJ. Effect of Delta-9-tetrahydrocannabinol and cannabidiol on nocturnal sleep and early-morning behavior in young adults. *J Clin Psychopharmacol*. 2004;24(3):305-313. doi:10.1097/01.jcp.0000125688.05091.8f

55. Nielsen S, Sabioni P, Trigo JM, et al. Opioid-Sparing Effect of Cannabinoids: A Systematic Review and Meta-Analysis. *Neuropsychopharmacology*. 2017;42(9):1752-1765. doi:10.1038/npp.2017.51

56. Feingold D, Brill S, Goor-Aryeh I, Delayahu Y, Lev-Ran S. Depression and anxiety among chronic pain patients receiving prescription opioids and medical marijuana. *J Affect Disord*. 2017;218:1-7. doi:10.1016/j.jad.2017.04.026
