# Supplementary figures and images for: Clinical Practice Guidelines for Cannabis and Cannabinoid-Based Medicines in the Management of Chronic Pain and Co-Occurring Conditions
Source: Cannabis Cannabinoid Res. 2024 Apr 1;9(2):669–87. doi: 10.1089/can.2021.0156 (PMC10998028; doi:10.1089/can.2021.0156)

Appendix A: PRISMA Flowchart


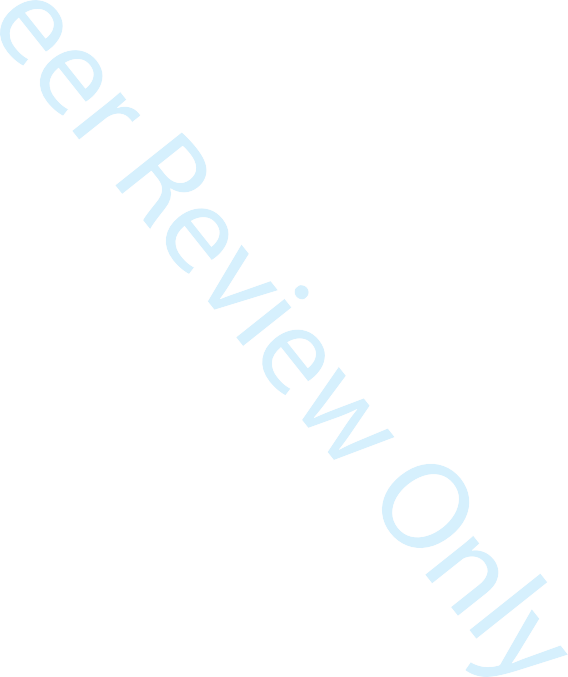

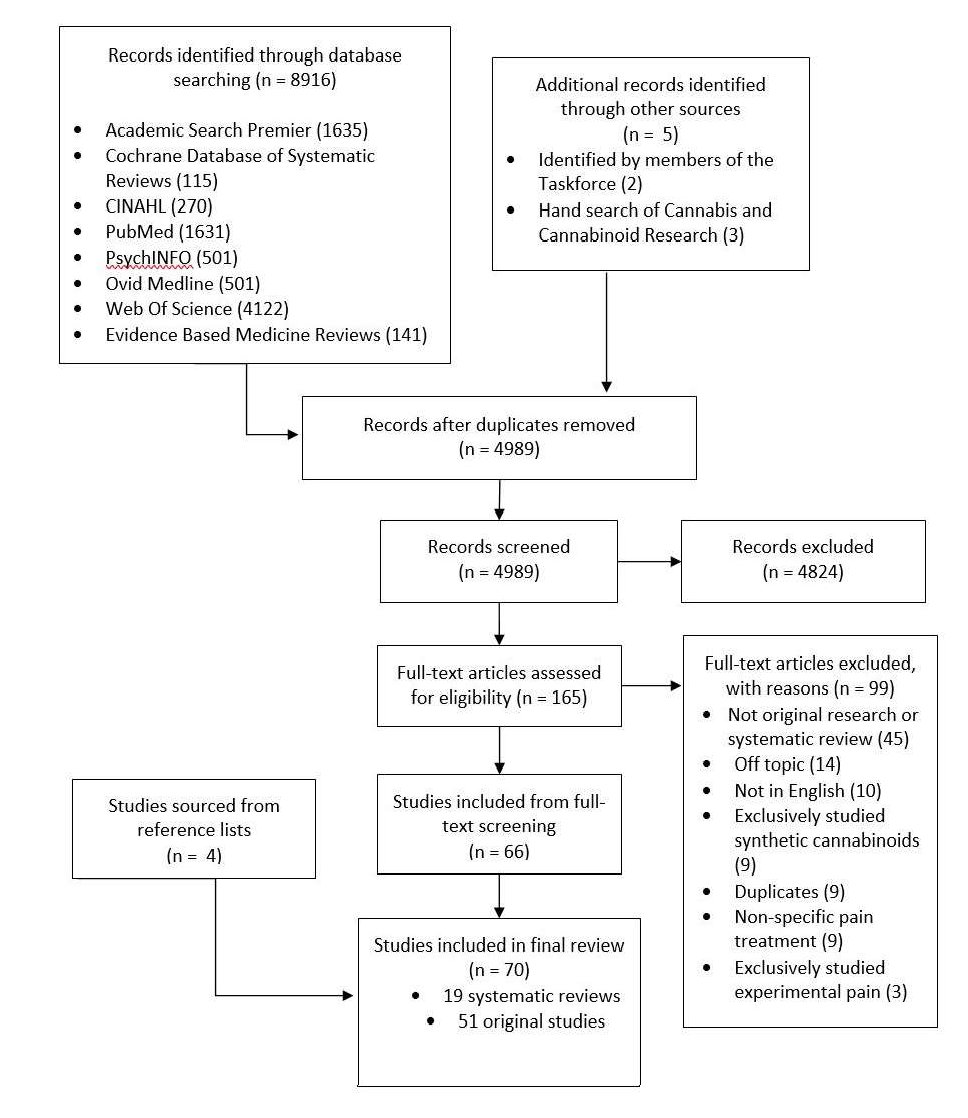

Supplement: Supplemental data [file Suppl_AppendixSA1.docx]
