# Supplementary material for: Clinical Practice Guidelines for Cannabis and Cannabinoid-Based Medicines in the Management of Chronic Pain and Co-Occurring Conditions
Source: Cannabis Cannabinoid Res. 2024 Apr 1;9(2):669–87. doi: 10.1089/can.2021.0156 (PMC10998028; doi:10.1089/can.2021.0156)
Supplement: Supplemental data [file Suppl_AppendixSA2.docx]

Appendix B: Summary of Previous Systematic Reviews

| **Reference** | **Number of studies included** | **Patient population** | **Cannabinoid based medicines included** | **Conclusions regarding efficacy** | **Quality Rating*** |
| --- | --- | --- | --- | --- | --- |
| **Allan et al. (2018)** | 31 | Pain, spasticity, or nausea and vomiting | Synthetic and non-synthetic cannabinoids | Insufficient evidence | Fair |
| **Andreae et al. (2015)** | 5 | Chronic neuropathic pain | Non-synthetic | Beneficial | Good |
| **Aviram & Samuelly-Leichtag (2017)** | 43 | Chronic or post-operative pain | Synthetic and non-synthetic cannabinoids | Beneficial | Fair |
| **Boychuk et al. (2015)** | 13 | Chronic non-cancer neuropathic pain | Synthetic and non-synthetic cannabinoids | Beneficial | Fair |
| **Deshpande et al. (2015)** | 6 | Chronic non-cancer pain | Non-synthetic | Beneficial | Good |
| **Fitzcharles et al. (2016)** | 4 | Rheumatic disease-associated chronic pain | Synthetic and non-synthetic cannabinoids | Insufficient evidence | Fair |
| **Hauser et al. (2017)** | 11 | Pain and palliative care | Synthetic and non-synthetic cannabinoids | Insufficient evidence | Fair |
| **Hauser et al. (2018)** | 10 | Chronic pain | Synthetic and non-synthetic cannabinoids | Insufficient evidence | Good |
| **Iskedjian et al. (2007)** | 7 | MS or comparable neuropathic pain | Synthetic and non-synthetic cannabinoids | Beneficial | Fair |
| **Lynch & Campbell (2011)** | 18 | Chronic non-cancer pain | Synthetic and non-synthetic cannabinoids | Beneficial | Fair |
| **Lynch & Ware (2015)** | 11 | Chronic non-cancer pain | Synthetic and non-synthetic cannabinoids | Beneficial | Good |
| **Madden et al. (2018)** | 118 | Non-cancer pain | Synthetic and non-synthetic cannabinoids | Insufficient evidence | Fair |
| **Martin-Sanchez et al. (2009)** | 18 | Pathological or traumatic chronic pain | Synthetic and non-synthetic cannabinoids (containing THC or a synthetic derivative) | Beneficial | Fair |
| **Meng et al. (2017)** | 11 | Chronic neuropathic pain | Synthetic and non-synthetic cannabinoids | Beneficial | Good |
| **Mucke et al. (2018)** | 16 | Chronic neuropathic pain | Synthetic and non-synthetic cannabinoids | Beneficial | Good |
| **Nugent et al. (2017)** | 75 | Chronic pain | Non-synthetic | Beneficial for neuropathic pain, insufficient evidence in other pain populations | Fair |
| **Park et al. (2017)** | 25 | Medical marijuana patients | Medical marijuana | Beneficial | Poor |
| **Stockings et al. (2018)** | 91 | Chronic non-cancer pain | Synthetic and non-synthetic cannabinoids | Insufficient evidence, nabiximols beneficial in MS-related pain | Good |
| **Whiting et al. (2015)** | 79 | Users of cannabinoids for a variety of specified indications | Synthetic and non-synthetic cannabinoids | Beneficial | Good |

*Quality ratings based on the NHLBI Study Quality Assessment Tools. A study with a ‘good’ score has the least risk of bias. A ‘fair’ study has a moderate risk of bias and a ‘poor’ study has significant risk of bias.
