# Supplementary material for: Clinical Practice Guidelines for Cannabis and Cannabinoid-Based Medicines in the Management of Chronic Pain and Co-Occurring Conditions
Source: Cannabis Cannabinoid Res. 2024 Apr 1;9(2):669–87. doi: 10.1089/can.2021.0156 (PMC10998028; doi:10.1089/can.2021.0156)
Supplement: Supplemental data [file Suppl_AppendixSA3.docx]

Appendix C: Summary of Included Primary Research Studies

| **Reference** | **Study Design** | **Sample Size** | **Patient Population** | **CBM Included** | **Length of Study** | **Pain Efficacy Conclusion** |
| --- | --- | --- | --- | --- | --- | --- |
| **Abrams et al. (2007)** | RCT | 50 | HIV | Smoked cannabis (3.56% THC) | 5 days | Beneficial |
| **Berman et al. (2004)** | RCT, three period crossover | 45 | Brachial plexus root avulsion patients | Cannabis extract oromucosal sprays | 14-20 days per treatment | Beneficial |
| **Blake et al. (2006)** | RCT | 58 | Rheumatoid arthritis | Nabiximols oromucosal spray | 5 weeks | Beneficial |
| **Collin et al. (2010)** | RCT | 337 | MS | Nabiximols oromucosal spray | 14 week | Nonsignificant |
| **Corey-Bloom et al. (2012)** | RCT, crossover | 30 | MS | Smoked cannabis (4% THC) | 3 days | Beneficial |
| **Ellis et al. (2009)** | RCT, crossover | 28 | HIV | Smoked cannabis | 5 days | Beneficial |
| **Hunter et al. 2018** | RCT | 320 | Osteoarthritis | Transdermal CBD | 12 weeks | Mixed results |
| **Langford et al. (2012)** | RCT | 339 | MS | Nabiximols oromucosal spray | 14 weeks | Mixed Results |
| **Notcutt et al. (2004)** | Series of N of 1 RCTS, crossover | 34 | Chronic pain patients | Cannabis extract oromucosal sprays | 8 weeks | Beneficial |
| **Novotna et al. (2011)** | RCT | 572 | MS | Nabiximols oromucosal spray | 19 weeks | Nonsignificant |
| **Nurmikko et al. (2007)** | RCT | 125 | Neuropathic pain and allodynia | Nabiximols oromucosal spray | 5 weeks | Beneficial |
| **Rog et al. (2005)** | RCT | 66 | MS | Nabiximols oromucosal spray | 5 weeks | Beneficial |
| **Selvarajah et al. (2010)** | RCT | 30 | Diabetes | Nabiximols oromucosal spray | 12 weeks | No evidence of pain efficacy |
| **Serpell et al. (2014)** | RCT | 246 | Peripheral neuropathic pain patients | Nabiximols oromucosal spray | 14 weeks | Beneficial |
| **Wade et al. (2003)** | Series of RCTs, crossover | 24 | Various neurological conditions | Cannabis extract oromucosal sprays | Two weeks per treatment | Beneficial |
| **Wade et al. (2004)** | RCT | 160 | MS | Nabiximols oromucosal spray | 6 weeks | No evidence of pain efficacy |
| **Ware et al. (2010)** | RCT, crossover | 21 | Chronic Neuropathic pain | Smoked cannabis (0%, 2.5%, 6% and 9.4% THC) | 5 days per treatment | Beneficial |
| **Weizman et al. (2018)** | RCT, crossover | 15 | Chronic radicular neuropathic pain | THC oil (0.2mg/kg, average dosage 15.4 +/- 2.2 mg) | Single dose with each treatment | Beneficial |
| **Wilsey et al. (2013)** | RCT, crossover | 39 | Neuropathic pain | Vaporized cannabis (1.29%THC, 3.53% THC) | Immediate effects measured | Beneficial |
| **Wilsey et al. (2016)** | RCT, crossover | 42 | Neuropathic pain from spinal cord injury or disease | Vaporized cannabis (2.9%THC, 6.7%THC) | Immediate effects measured | Beneficial |
| **Wilsey et al. (2008)** | RCT, crossover | 38 | Neuropathic pain | Smoked cannabis (7%THC, 3.5%THC) | Immediate effects measured | Beneficial |
| **Zajicek et al. (2012)** | RCT | 279 | MS | Cannabis extract capsules (2.5mg THC) | 12 weeks | Beneficial |
| **Zajicek et al. (2005)** | RCT | 502 | MS | Cannabis extract capsules | 1 year | Beneficial |
| **Zajicek et al. (2003)** | RCT | 630 | MS | Cannabis extract capsules (2.5mgTHC, 1.25mgCBD) | 14 weeks | Beneficial |
| **Bonn-Miller et al. (2014)** | Cross-sectional | 217 | Medical cannabis patients | Cannabis | N/A | Beneficial |
| **Brunt et al. (2014)** | Cross-sectional | 113 | Medical cannabis patients | Cannabis (High, medium and low THC) | N/A | Beneficial |
| **Campbell et al. (2018)** | Prospective cohort | 1514 | Chronic pain patients prescribed opioids | Cannabis | 4 years | Mixed |
| **Clark et al. (2004)** | Cross-sectional | 220 | MS | Cannabis | N/A | Beneficial |
| **Degenhardt et al. (2015)** | Cross-sectional | 1514 | Opioid-prescribed pain patients | Cannabis | N/A | Beneficial |
| **Fanelli et al. (2017)** | Retrospective case-series analysis | 614 | Chronic pain | Cannabis | Mean follow up at 98.4 days | Beneficial |
| **Habib & Artul (2018)** | Retrospective Review | 26 | Fibromyalgia | Medical Cannabis | Median duration of 3 months | Beneficial |
| **Nugent et al. (2018)** | Cross-sectional | 371 | Pain patients on long-term opioid therapy | Medical cannabis | N/A | Beneficial |
| **Rhyne et al. (2016)** | Retrospective chart review | 121 | Chronic headache | Medical cannabis | N/A | Beneficial |
| **Tripp et al. (2014)** | Outpatient survey | 342 | Men with chronic prostatitis/chronic pelvic pain syndrome | Cannabis | N/A | Inconclusive |
| **Vigil et al. (2017)** | Cohort study | 66 | Chronic pain patients prescribed opioids | Cannabis products | 19-21 months | Beneficial |
| **Ware et al. (2003)** | Prospective cross-sectional | 209 | Chronic pain | Medicinal cannabis | N/A | Beneficial |
| **Woolridge et al. (2005)** | Cross-sectional | 523 | HIV | Cannabis | N/A | Beneficial |
| **Yassin et al. (2019)** | Observational cross-over | 31 | Fibromyalgia | Cannabis | 6 months minimum | Beneficial |
| **Abrams et al. (2011)** | 2 arm intervention | 21 | Chronic pain, prescribed opioids | Vaporized cannabis | 5 days | Beneficial |
| **Bellnier et al. (2018)** | Retrospective, mirror-image | 29 | Chronic pain | Medical cannabis | 3 months | Beneficial |
| **Chan et al. (2017)** | Pre/post | 588 | PTSD | Medical cannabis | 10 months | Beneficial |
| **Cuñetti et al. (2017)** | No control intervention | 7 | Kidney transplant patients | CBD | 3 weeks | Insufficient evidence |
| **Haroutounian et al. (2008)** | open-label add on | 13 | Chronic pain | THC | Treatment range of 2 weeks to 36 months | Insufficient evidence |
| **Haroutounian et al. (2016)** | Prospective open-label | 206 | Chronic pain | Cannabis | 6 months | Beneficial |
| **Hoggart et al. (2015)** | Open-label, follow on | 380 | Neuropathic pain | Nabiximols oromucosal spray | 38 weeks | Beneficial |
| **Nicolodi et al. (2017)** | Test-Retest | 48 | Chronic migraine | THC+CBD (200mg/day) | 3 month | Beneficial |
| **Poli et al. (2018)** | Non randomized, single arm trial | 338 | Chronic pain | Cannabis, prepared as tea | 1 year | Beneficial |
| **Rog et al. (2007)** | Open-label extension trial | 63 | MS | Nabiximols oromucosal spray | 2 years | Beneficial |
| **Russo et al. (2016)** | Pre/post | 20 | MS | Nabiximols oromucosal spray | 4 weeks | Beneficial |
| **Lynch et al. (2006)** | Case-series | 30 | Chronic pain | Medical cannabis | N/A | Beneficial |
| **Ware et al. (2002)** | Case-series | 15 | Chronic pain | Cannabis | N/A | Beneficial |
